# Fully Automated and AI-Assisted Optical Fiber Sensing System for Multiplexed and Continuous Brain Monitoring

**DOI:** 10.1021/acssensors.4c02126

**Published:** 2024-12-04

**Authors:** Yuqian Zhang, Naihan Zhang, Yubing Hu, Christopher Pereira, Michael Fertleman, Nan Jiang, Ali K. Yetisen

**Affiliations:** † Department of Chemical Engineering, Imperial College London, London SW7 2AZ, U.K.; ‡ Institute of Lightwave Technology, Ministry of Education, Beijing Jiaotong University, Beijing 100044, China; § Cutrale Perioperative and Ageing Group, Department of Bioengineering, Imperial College London, London W12 0BZ, U.K.; ∥ West China School of Basic Medical Sciences & Forensic Medicine, Sichuan University, Chengdu 610041, China; ⊥ Jinfeng Laboratory, Chongqing 401329, China

**Keywords:** fiber optics, multiplexed sensing, machine learning modeling, fluorescent sensor, brain monitoring

## Abstract

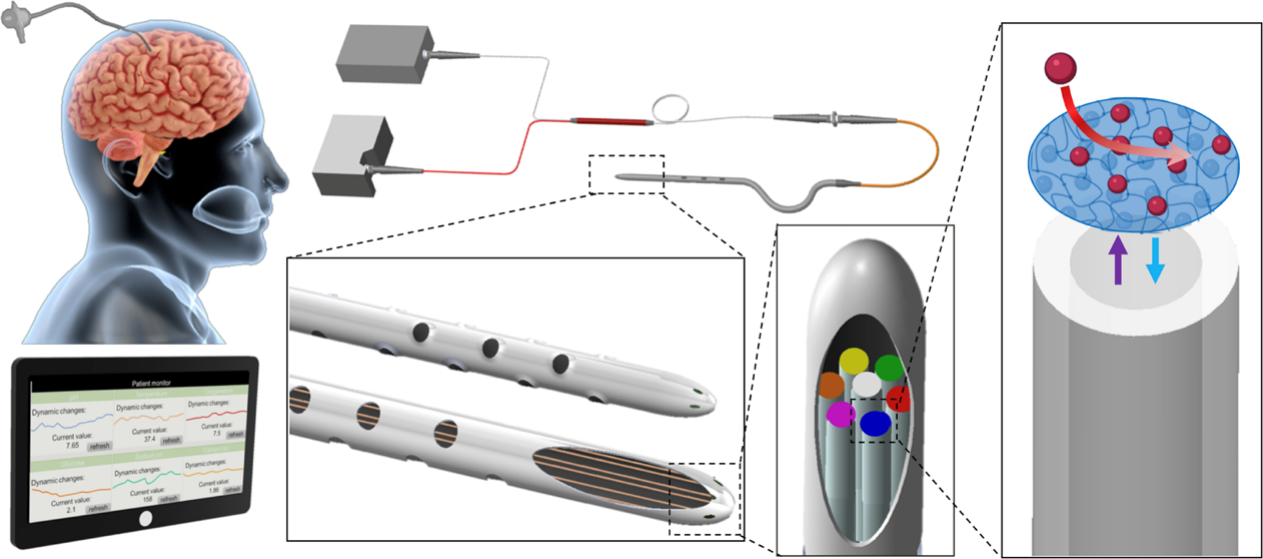

Continuous and comprehensive brain monitoring is crucial for timely identification of changes or deterioration in brain function, enabling prompt intervention and personalized treatments. However, existing brain monitoring systems struggle to offer continuous and accurate monitoring of multiple brain biomarkers simultaneously. This study introduces a multiplexed optical fiber sensing system for continuous and simultaneous monitoring of six cerebrospinal fluid (CSF) biomarkers using tip-functionalized optical fibers and computational algorithms. Optimized machine learning models are developed and integrated for real-time spectra analysis, allowing for precise and continuous readout of biomarker concentrations. The developed machine learning-assisted fiber optic sensing system exhibits high sensitivity (0.04, 0.38, 0.67, 2.62, 0.0064, 0.33 *I*/*I*_0_ change per units of temperature, dissolved oxygen, glucose, pH, Na^+^, Ca^2+^, respectively), reversibility, and selectivity toward target biomarkers with a total diameter less than 2.5 mm. By monitoring brain metabolic and ionic dynamics, this system accurately identified brain physiology deterioration and recovery using *ex vivo* traumatic brain injury models. Additionally, the system successfully tracked biomarker fluctuations in clinical CSF samples with high accuracy (*R*^2^ > 0.93), demonstrating excellent sensitivity and selectivity in reflecting disease progression in real time. These findings underscore the enormous potential of automated and multiplexed optical fiber sensing systems for intraoperative and postoperative monitoring of brain physiologies.

Real-time clinical and bedside monitoring of key physiological biomarkers plays a pivotal role in medical diagnostics and treatment. These monitoring practices provide invaluable insights into physical functions, enabling healthcare professionals to effectively diagnose and manage various disorders. In the intensive care unit (ICU) and other healthcare settings, brain monitoring serves as a critical component of patient care, aiding in the early detection of neurological abnormalities, monitoring the progression of brain injuries,^1,2^ guiding treatment decisions,^2,3^ and assessing the efficacy of therapeutic interventions.^4^ In clinics, traditional monitoring techniques, such as electroencephalography (EEG),^5^ intracranial pressure (ICP) monitoring,^2,6^ cerebral oximetry,^1,7^ microdialysis,^1,7^ and cerebral blood flow assessment,^1,7^ have significantly aided clinicians in clinical decisions-making and interventions. In recent years, more and more studies have been published for continuous and multiple brain biomarkers monitoring with various sensing modalities.^2,3,8^ However, current challenges remain in insufficient temporal resolution and limited biomarker numbers and types that can be monitored, which may hinder continuous and prolonged monitoring, limiting clinician’s ability to obtain comprehensive and accurate information about a patient’s brain health.

In recent years, significant advances in general engineering technologies, such as miniaturization, nano/micro scale fabrication, and low-power consumption, have enabled the development of compact and portable monitoring devices that can be easily integrated into clinical workflows and bedside settings.^8−10^ Additionally, optical fiber-based sensing techniques have emerged as promising modalities for minimally invasive and deep tissue measurement of biomarkers with high sensing performance.^10−12^ These optical fiber sensing techniques leverage the interaction of light with biological tissues to measure changes in cerebral blood flow, oxygenation levels, and neuronal activation patterns.^10,12^ Real-time and continuous monitoring of these biomarkers holds significant promise for intraoperative and postoperative tracking of brain physiology status, providing crucial insights into brain health and aiding in clinical diagnosis and treatment. However, to date, most proposed optical fiber sensing systems can only offer single biomarker monitoring, whereas, in clinical settings, continuous monitoring of several biomarkers is more desirable for precise and accurate disease diagnosis and treatment.^10,13^ While we have previously developed a multiplexed sensing system to detect 4 biomarkers in artificial cerebrospinal fluid (CSF) by functionalizing different sensing probes on a single reflection optic fiber tip, it suffers from several challenges and limitations due to the design of the system.^13^ The multiplexing was realized using 2-channel reflection fiber with the sensing films placed in front of the fiber tip through a silicone shaft, leading to large sensor dimension (8 mm), unrepeatable fabrication, severe spectra overlapping, and limited sensing capability (up to 4 biomarkers). However, by leveraging the single-channel fluorescent sensing bundle, more biomarkers can be measured simultaneously with easier fabrication methods, higher stability, and lower probe dimension.

In spectroscopy-based optical sensing, accurately determining biomarker concentrations from measured optical signals poses a significant challenge due to factors such as background noise, low sampling rates, and spectral overlap. Computational methods, ranging from basic statistic modeling to more advanced algorithms such as machine learning, have been integrated with optical sensors and immunoassays to overcome the chemical limitation and provide highly accurate and better-performed biomarker monitoring.^14−19^ These algorithms have demonstrated high possibilities in providing accurate predictions of biomarker levels, signal separation, noise reduction, and diagnostic test. For instance, convolutional neural networks (CNN)-based decoding methods have been utilized with microfluidic and immunoassays to achieve multiplexed detection of inflammatory markers and antibiotics with high detection range and sensitivity.^16,18^ In another study, machine learning-based models were developed to precisely separate fluorescent spectra that enable a cost-effective fluorescence sensing setup for scalable and multiplexed biomarker detection.^19^ Furthermore, Gaussian process and multivariate classification modeling have been demonstrated to directly predict the oxygenation levels in patients with brain injury using optical oxygenation sensors for the prevention of secondary brain injury.^15^

Herein, we developed a fully automated multiplexed optical fiber sensing system based on artificial intelligence (AI)-integrated optical fiber bundle sensors to address challenges in multiplexed dynamic monitoring of six human cerebrospinal fluid (CSF) biomarkers. Brain biomarkers—temperature (*T*), dissolved oxygen (DO), pH, sodium ions (Na^+^), calcium ions (Ca^2+^), and glucose—were selected as targets for their relevance to various brain disorders and sensitivity in reflecting brain physiology status.^20−25^ Initially, an optical fiber bundle was constructed by bundling seven single optical silica fibers as a sensing substrate integrated into a CSF drainage catheter for measurements. The tips of the fiber bundle were functionalized with six fluorescent sensing films as reversible optical transducers, enabling the selective and continuous measurement of the corresponding biomarkers. Additionally, a spare optical fiber was included for potential extension or signal enhancement. AI-assisted postsignal processing algorithms were developed for spectrum processing and concentration prediction, complemented by a graphical UI for direct user readout. The validity of the sensing system was evaluated using *ex vivo* brain models and clinical human CSF samples, simulating brain injury physiologies/complications in traumatic brain injury (TBI) patients. The developed multiplexed optical fiber sensing system demonstrated excellent performance in continuous and quantitative monitoring of multiple brain biomarkers in the CSF in real time. Each optical fiber sensor in the bundle exhibited high sensitivity and selectivity toward its target analyte. Efficient and robust computer software with AI models successfully aided in controlling the measurement optical system, ensuring stable and precise biomarker readouts. The optical fiber multiplexed sensing system holds great potential in assisting clinicians with dynamic monitoring of brain physiology in bedside settings.

## Results

### Design and Overview of the Multiplexed Optical Fiber Sensing System

**Figure 1:**
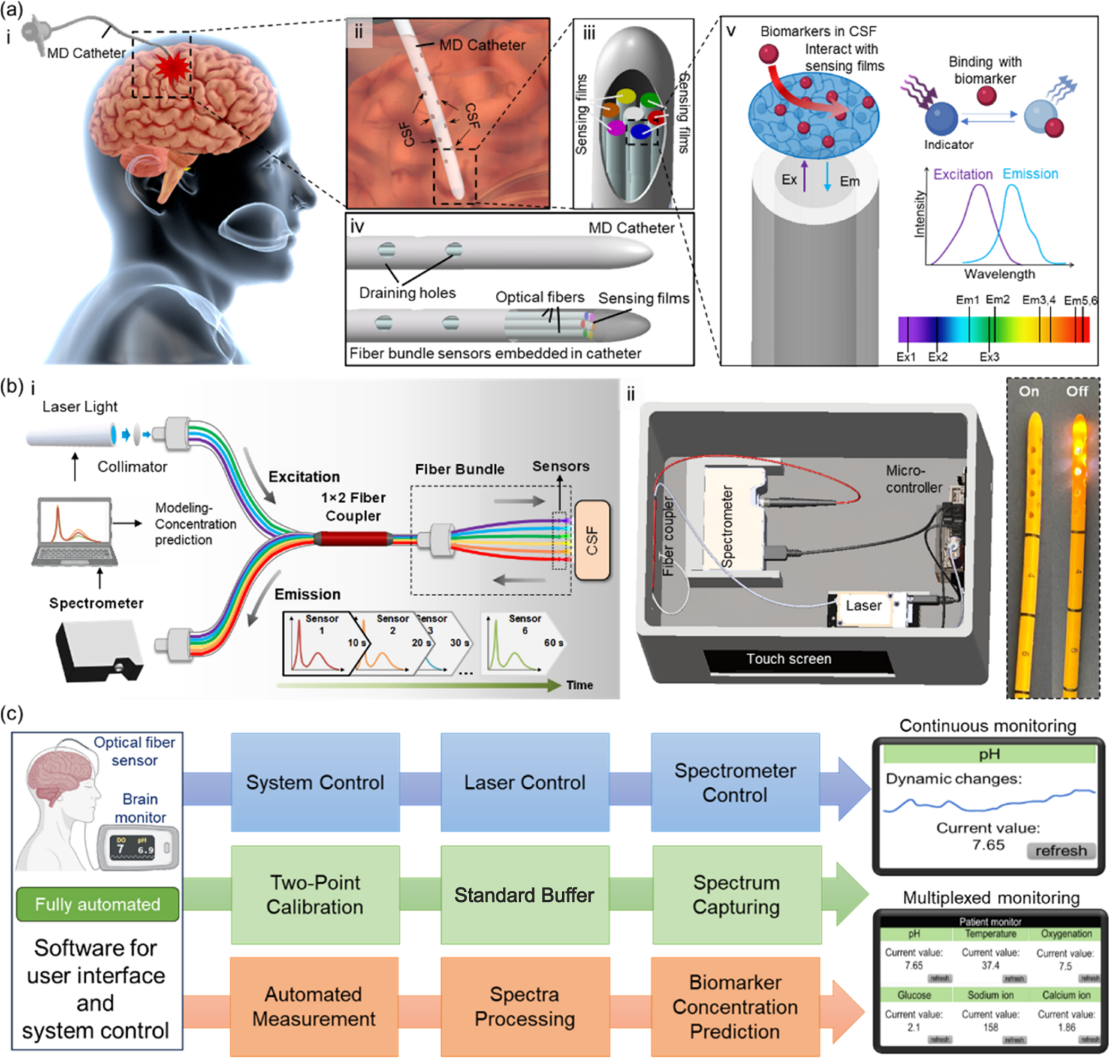
Optical fiber bundle sensing system for multiplexed biomarker monitoring in brain CSF. (a) (i) Schematic of the designed bundled optical fiber sensor integrated with brain catheter for multiplexed CSF biomarker monitoring. (ii) The designed bundle and the catheter are inserted into brain tissue for CSF collection and biomarker monitoring. (iii) Zoomed-in figure of the self-designed optical bundle with 6 fluorescent sensing films on the fiber tips inside the catheter. (iv) Skeleton view of the catheter and the fiber bundle. (v) Fluorescent probes encapsulated in polymer films on fiber tips for selective measurement of biomarkers. The fluorescent probes have either different excitation or emission wavelength. (b) (i) Schematic and (ii) photograph of the optical waveguides and sensing system. (c) Software for the development of a brain monitor, which includes three parts: hardware control module, sensor calibration module, and measurement and readout module.

In this study, we developed an optical fiber bundle sensing system designed for the simultaneous measurement of six brain biomarkers. A flexible silicone elastomer biofluid extraction catheter (outer diameter: 2.5 mm, inner diameter: 1.3 mm) is integrated with the sensing bundle, enabling both cerebrospinal fluid (CSF) collection and real-time measurement of the six biomarkers with minimized brain damage and surgical procedures (Figure 1a). The incorporated sensing catheter is intended for insertion into the brain of traumatic brain injury (TBI) patients, facilitating multiplexed and dynamic brain CSF monitoring (Figure 1a-i,ii). The measurement probe for optical fiber sensing consists of a bundle comprising seven evenly distributed optical fiber sensors. One end is connected with a fixed connector, while the other ends are inserted into the brain drainage catheter (Figures 1a, S1, and S2). On the optical fiber tips, six different fluorescent sensing films are individually functionalized by encapsulating fluorescent indicators in polymer films (Figure 1a-iii). Brain CSF is extracted through the catheter holes, allowing for dynamic monitoring of the six different biomarkers in the collected CSF sample using the optical fiber sensors inside the catheter (Figure 1a-iv). Changes in the emission intensity of the fluorescent sensors, upon interaction with the target biomarkers, reflect the biomarker concentrations in CSF continuously and in real time (Figure 1a-v).

Typically, each optical fiber sensor necessitates one spectrometer and one laser/LED for measurement, resulting in the requirement of six lasers and six spectrometers for monitoring the six biomarkers. However, our design optimizes the system by incorporating 6 optical fiber sensors with either different excitation or emission wavelengths, bundled together. This reduces the requirement to only one spectrometer and one switchable multiwavelength laser source for monitoring all six biomarkers. The connection between the sensor bundle, laser light source, and the spectrometer is facilitated through an optical coupler (Figure 1b). The multiwavelength laser, offering outputs at three different wavelengths (405, 488, and 520 nm), can be programmably controlled to excite the six fluorescent optical fiber sensors. The corresponding emission spectra are collected by the spectrometer under different laser excitations. Signal processing algorithms are then applied to analyze the obtained spectra and derive accurate and precise brain biomarker concentrations for clinical references. The developed optical sensing system (Figure 1b-ii) showcases the optical sensing bundle inserted into the medical catheter, featuring a lightweight design that is user-friendly and easy to operate.

The software orchestrates complete system control and incorporates a user-friendly graphical interface (UI) for seamless control and data visualization. Its functionalities encompass three core aspects: First, it governs the lasers and spectrometer through UART communication. Second, it conducts sensor calibration before each measurement to ensure accuracy. Lastly, using AI models, it computes biomarker concentrations from acquired fluorescent spectra. Two readout methods are provided by the software, a dynamic readout for individual biomarkers, showcasing their real-time fluctuations; and a multiplexed readout, displaying concurrent concentrations of all six biomarkers. To ensure precise brain biomarker calculations, extensive exploration and comparison of machine learning algorithms were undertaken for integration into the brain monitoring system.

### Temperature Sensor Characterization

**Figure 2:**
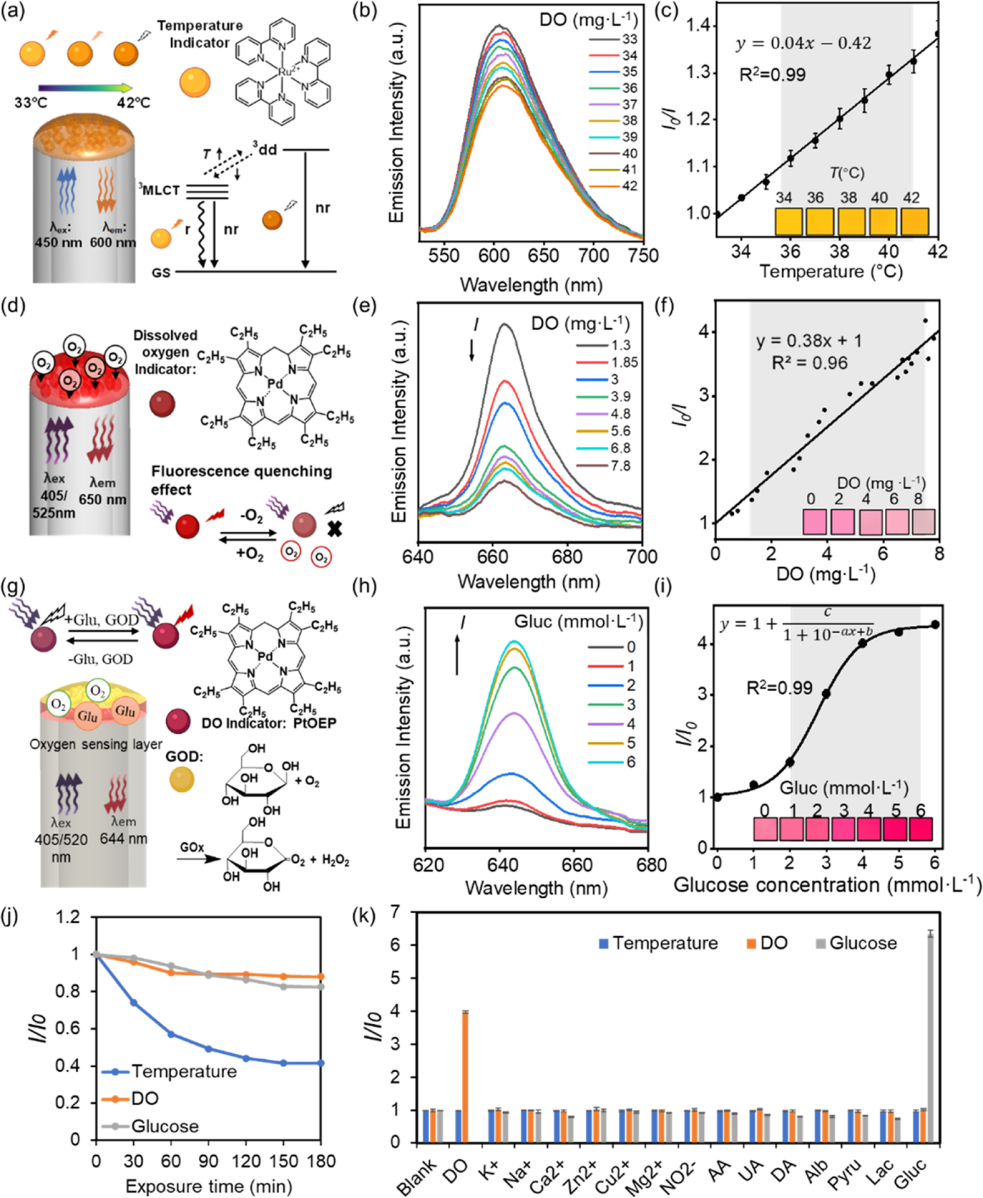
Design and characterization of the optical fiber sensor for temperature, DO, and glucose monitoring in PBS solutions (10 mmol L^–1^, pH 7.4). (a) Schematic demonstration of the temperature sensing principle. nr: nonradiative route. (b) Emission spectra of the optical fiber sensor for monitoring PBS solution with temperature of 33–42 °C. (c) The calibration curve of the temperature sensor for the sensing of temperature from 33 to 42 °C. Shadows represent the physiological range of temperature in human CSF. (d) Schematic demonstration of the design and principle of the poly(dimethylsiloxane)-palladium(II) octaethylporphine (PDMS-PdOEP) DO sensor. (e) Fluorescence spectra of the sensor in PBS solutions with different DO concentrations (1–8 mg L^–1^). (f) Calibration curve of the DO sensor for the measurement of 1–8 mg L^–1^ DO. (g) Schematic demonstration of the design and principle of the glucose-oxidase-PTOEP-Sol–Gel glucose sensor. (h) Fluorescence spectra of the glucose sensor in PBS solutions with different glucose concentrations (0–6 mmol L^–1^). (i) The calibration curve of the glucose sensor for the measurement of 0–6 mmol L^–1^ glucose. (j) Stability of the temperature, DO, and glucose sensor under continuous exposure to 405 nm (4.5 mW) laser light. (k) Selectivity test of the temperature, DO, and glucose sensors against major interferences (Table S1). Gluc: glucose; Lac: lactate; UA: uric acid; AA: ascorbic acid; DA: dopamine; Alb: albumin; Pyru: pyruvate. Error bars represent the standard deviation of the mean of the three measurements. Shadows represent the physiological range of biomarkers in the human CSF. *n* = 3.

Temperature is a fundamental biomarker in physiology, especially in patients with TBI, as it is closely associated with infection and metabolite variations.^20^ Tris(2,2′-bipyridyl)dichlororuthenium(II) hexahydrate (Ru(bpy)_3_) contains a ruthenium group known for its high sensitivity to temperature changes,^26^ whose fluorescence can be quenched at high temperature, where the ^3^MLCT state can be thermally populated to metal-localized triplet state (^3^dd), which subsequently decays to ground state via a nonradiative route,^27^ resulting in a decreased emission intensity (Figure 2a). However, Ru(bpy)_3_ is also sensitive to oxygen levels, and thus, an epoxy film with a relatively low air permeability (2–10 cm^3^ m^–2^ per 24 h) was used to encapsulate the fluorescent indicator, creating a temperature sensing film that minimizes oxygen interference. The temperature sensing film was fabricated on the tip of a single optical fiber by dip coating in an epoxy-Ethenol-Ru(dpy)3 pregel solution with optimized probe concentration (Supporting Information, Figure S3a). The single optical fiber sensor was then connected to a 450 nm laser light and a spectrometer via a fiber coupler. In the validation, an evident decreasing trend in the emission intensity of the Ru(bpy)_3_-containing epoxy-based temperature sensing film was observed as the phosphate-buffered saline (PBS) solution temperature increased from 33 to 42 °C to cover the physiological range (Figure 2b). A good linear relationship was established between the emission intensity ratio (*I*_0_/*I*) and temperature with a sensitivity of 0.04/°C (*R*^2^ = 0.99) (Figure 2c). The response of the temperature sensor is instant (<1 s) because the sensing mechanism is based on physical properties and no chemical reaction is involved.

### Dissolved Oxygen (DO) Sensor Characterization

DO is a critical brain physiology parameter that reflects tissue metabolism oxygenation in TBI patients.^21,28^ A lower DO indicates a high risk of brain hypoxia,^28^ while excessive oxygen supplementation could lead to oxygen toxicity, causing seizures and unconsciousness.^29^ For brain DO monitoring, a palladium(II) octaethylporphine (PdOEP)-encapsulated poly(dimethylsiloxane) (PDMS) sensing film was coated on an optical fiber tip for high oxygen permeability and low water permeability, which is benefit for high measurement sensitivity.^30,31^ The oxygen sensing principle is based on the fluorescence quenching effect in the presence of oxygen (Figure 2d), and the probe concentration was optimized to have a good intensity (Figure S3b). In the film characterization test, the oxygen sensing film demonstrated a high sensitivity toward oxygen with 0.38 increases in *I*_0_/*I* per oxygen unit. As the DO concentration increased from 0.68 to 7.98 mg·L^–1^, the emission intensity of the DO sensor was reduced by 85.6% (Figure 2e,2f). A maximum response time of 4 min was observed in measuring various DO levels.

### Glucose Sensor Characterization

The levels of glucose in the CSF are commonly measured in clinics to assess brain physiology in TBI patients due to its importance in brain metabolism.^25^ In the early stages of TBI, patients typically suffer from low oxygen and glucose supply, as brain blood flow is impaired. However, in the later stages, the glucose uptake has been observed to increase along with the metabolism.^25^ Therefore, measuring the glucose levels in brain tissue would indicate disease progression.^25^ To achieve dynamic and real-time glucose monitoring, a sensor with two sensing layers was constructed: an outer layer of glucose oxidase-immobilized polyacrylamide hydrogel for glucose redox reaction catalyzation with oxygen; and an inner layer of oxygen-sensitive probe platinum octaethylporphyrin (PtOEP) encapsulated in a sol–gel film for oxygen measurement (Figure 2g) for distinguishable peaks.^32^ The sensor was characterized by using PBS solutions with different glucose concentrations ranging from 0 to 6 mmol·L^–1^ (Figure 2h). During the experiment, the solution was stirred at a constant speed to ensure an air/oxygen-saturated solution throughout the measurement to minimize oxygen influences. There is an equilibrium between the speed of oxygen consumption and the oxygen supply, and thus all detection ranges for glucose can be designed by optimizing the glucose oxidase concentration during sensor fabrication. The emission intensity of the glucose sensor increased as glucose concentration rose, with a correlation between glucose concentration and emission intensity fitting a sigmoid curve (*R*^2^ = 0.99), resulting in a 4-fold signal increase from 1 to 5 mmol·L^–1^ of glucose (Figure 2i). The glucose sensor response time is related to the enzyme concentration and for this current design, the longest response time is 4 min.

In addition to sensitivity, the sensors underwent thorough characterization for stability, reversibility, environmental robustness, and selectivity, which are essential for sustained clinical applications. All three sensors (temperature, DO, and glucose) exhibited high reversibility (Figures S4–S6), indicating their capability to detect both increases and decreases in the target biomarker levels. This robust reversibility ensures dependable readouts during continuous measurement. For continuous monitoring, the photostability is crucial. Following a 3-h continuous exposure to respective excitation laser lights (at 4.5 mW), the temperature sensor’s emission intensity decreased by 60%, while the DO sensor retained 87% intensity, and the glucose sensor maintained 80% intensity (Figure 2j). Although these decreases stem from inevitable photobleaching effects of fluorescent-based probes, mitigating strategies such as employing lower power lasers, pulsed lasers, and computational compensation methods may be necessary to reduce photobleaching in continuous applications. Assessments were made regarding the environmental robustness and selectivity. All three sensors demonstrated consistency across various pressure and pH levels (Figures S4–S6). The reduction in intensities with rising temperature is attributed to temperature-induced quenching effects on the fluorescent probes (Figures S4–S6), correctable in postsignal processing based on temperature sensor readings. Furthermore, the sensors exhibited high selectivity, showing minimal variation in spectra when exposed to interference in the sensing solution. This observation underscores their potential for precise temperature, DO, and glucose measurements in real clinical scenarios (Figure 2k).

### pH Sensor Characterization

**Figure 3:**
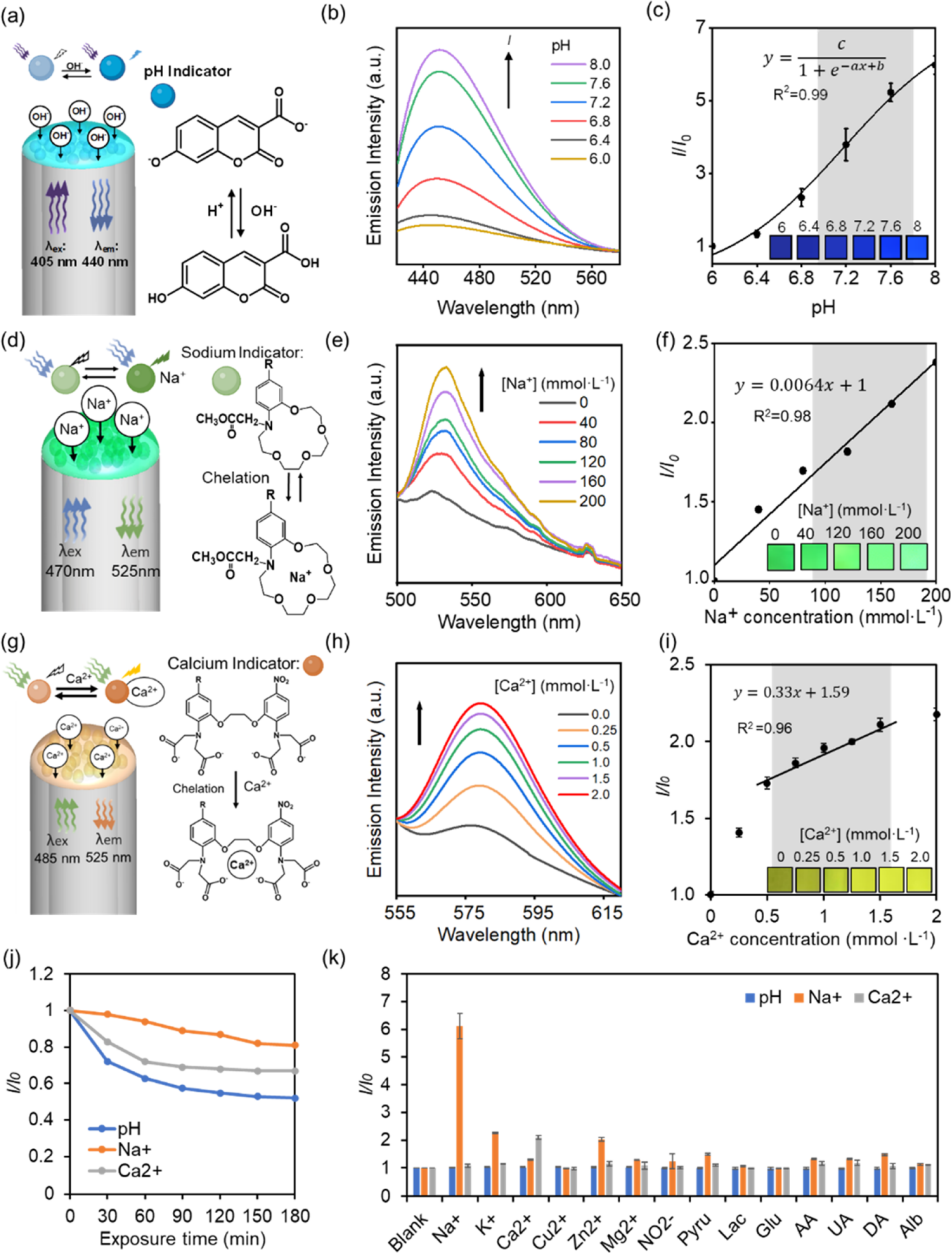
Design and characterization of the pH, Na^+^, and Ca^2+^ sensor in standard buffer solutions (10 mmol L^–1^, pH 7.4, 25 °C). (a) Schematic of the optical fiber HCC-sol–gel pH sensor design and sensing principle. (b) Fluorescence spectra of the sensor dipped in pH 6.0–8.0 PBS solutions. (c) The calibration curve of the pH sensor for the measurement of pH 6.0–8.0 PBS solution. (d) Schematic drawing of the Corona Green-PEGDA Na^+^ sensor and sensing principle. R: fluorescein. (e) Fluorescence spectra of the Na^+^ sensor in 0–200 mmol L^–1^ [Na^+^] Tris buffer solution. (f) The calibration curve of the Na^+^ sensor for the measurement of 0–200 mmol L^–1^ [Na^+^] Tris buffer solution. (g) Schematic drawing of the Rhod5N-pAM Ca^2+^ ion sensor and sensing principle. (h) Fluorescence spectra of the Ca^2+^ ion sensor for the measurement of 0–2 mmol L^–1^ [Ca^2+^] Tris buffer solution. (i) The calibration curve of the Ca^2+^ ion sensor for the measurement of 0–200 mmol L^–1^ [Ca^2+^] Tris buffer solution. (i) Photostability of the sensors under continuous exposure of 405 nm (pH sensor) 450 nm (Na^+^ sensor) and 520 nm (Ca^2+^ sensor) lasers for 3 h. (k) Selectivity test of the sensor of measuring standard buffer solution against major interferences (Supporting Information, Table S1). UA: uric acid; AA: ascorbic acid; DA: dopamine; Alb: albumin. Pyru: pyruvate; Lac: lactate; Gluc: glucose. Error bars represent the standard deviation of the mean of three measurements. Shadows represent the physiological range of biomarkers in human CSF. *n* = 3.

Regulation of brain pH is a vital homeostatic function in the central nervous system, and its association with the severity and clinical outcome of TBI patients makes it an important clinical parameter.^22^ Here, fluorophore 7-hydroxycoumarin-3-carboxylic acid (HCC) was used as the pH indicator and encapsulated in proton-permeable sol–gel films with optimized concentrations (Supporting Information, Figure S3c). The pH sensing process of HCC in PBS solution of pH 6 to 8 involves two deprotonation steps: first, the deprotonation of imino group (p*K*_a_ = 2.7), and then the deprotonation of phenol group (p*K*_a_ = 7.8), causing the maximum excitation wavelength of HCC to shift from 352 to 385 nm, and a total p*K*_a_ of 7.0 for HCC is obtained.^33,34^ Therefore, if the excitation wavelength is set to be >385 nm, the emission intensity would continually increase as pH increases (Figure 3a). In this study, a 405 nm laser light was used to excite the pH optical fiber sensor, and the emission intensity at 450 nm was correlated with the pH value. The results indicated a sensitivity of 2.624 *I*/*I*_0_ per pH unit, and the emission intensity increases 5 times within 3 min as the pH value increased from 6.0 to 8.0, with the calibration curve fitting a sigmoidal model (Figure 3b,c).

### Na^+^ Sensor Characterization

In patients with TBI, sodium influx is commonly observed due to increased glutamate concentration, leading to sodium-dependent neuronal swelling and excitotoxicity.^24^ Therefore, continuous and real-time measurement of the temporal changes in Na^+^ ion concentrations is of great significance in reflecting brain physiology. In this study, CoroNa Green probe, which selectively binds with Na^+^ ion in the human physiological range (*K*_d_ of ∼80 mmol·L^–1^), was utilized to fabricate the optical fiber sodium sensor by encapsulation in a poly(ethylene glycol) diacrylate-acrylamide (PEGDA-AM) hydrogel (Figure 3d).^35^ The probe comprises a fluorescein molecule and a crown ether, which has a cavity size selective for Na^+^ ions, exhibiting increased fluorescence emission intensity upon binding with the Na^+^ ion. The probe concentration was optimized for the fabrication of the sensing film (Supporting Information, Figure S3d), and the PEGDA-AM hydrogel was chosen due to its high biocompatibility and cation permeability. In the sensitivity test, the sodium sensing film exhibited a high sensitivity of 0.064 *I*/*I*_0_ per change of 10 mmol·L^–1^ in the sodium ion concentration ([Na^+^]) across the whole range from 0 to 200 mmol·L^–1^ of [Na^+^] with good linearity (*R*^2^ = 0.98) (Figure 3e,f). A fast response time of less than 2 min was observed after switching to 200 mmol·L^–1^ buffer solution from 0 mmol·L^–1^.

### Ca^2+^ Sensor Characterization

Ca^2+^ ions play an important role in cell development, neurotransmissions, and learning,^23^ and the maintenance of Ca^2+^ ions and their homeostasis are essential for the function of the nervous system. After TBI, elevated Ca^2+^ ion concentration ([Ca^2+^]) is commonly observed in brain CSF, caused by brain ischemia due to reduced cerebral blood flow and elevated glutamate levels, leading to secondary brain injury.^23^ Here, [Ca^2+^] was detected by encapsulating Rhod-5N, which consists of a 1,2 bis(*o*-aminophenoxy) ethane-*N*,*N*,-*N*′,*N*′-tetraacetic acid (BAPTA) moiety for Ca^2+^ ion recognition and fluorophore rhodamine, in a polyacrylamide-MBA hydrogel (Figures 3g and S10). The BAPTA moiety selectively binds with Ca^2+^ ions with a high *K*_d_ of 0.5 mmol L^–1^, ensuring a reversible response and a large detection range.^36^ The emission intensity increased proportionally with [Ca^2+^]. The encapsulation concentration of the calcium indicator and the hydrogel fabrication method were optimized to achieve an optimal signal-to-noise ratio (Supporting Information, Figure S2, Figure S3e). The calcium sensor’s characterization was conducted by dipping in tris-calcium buffer solutions and exposure to a 520 nm laser as the excitation source. The results showed that the fabricated calcium sensor had a sensitivity of 0.41 increase in *I*/*I*_0_ per Ca^2+^ unit in physiological range (Figure 3h) and demonstrated a sigmoid correlation with *R*^2^ = 0.98 and a linear correlation within the physiological range (*R*^2^ = 0.96, Figure 3i). The response time of the calcium ion sensor is less than 3 min.

These three cation sensors also underwent comprehensive testing to evaluate their photostability, reversibility, environmental robustness, and selectivity (Figures 3j,k and S7–S9). When subjected to continuous exposure under their respective optimal excitation laser lights, the pH sensor retained 48% of its initial emission intensity after 3 h. Similarly, the sodium sensor maintained 62% of its initial intensity, while the calcium sensor experienced a 35% decrease due to fluorophore photobleaching, potentially impacting biomarker concentration calculations (Figure 3j). To ensure accuracy in long-term measurements, alternative strategies are imperative. However, these cation sensors demonstrated a remarkable ability to measure elevated cation levels, evident through larger *I*/*I*_0_ values, and exhibited recovery to their original state in lower ion samples during repeated testing, signifying a high reversibility crucial for reliable continuous monitoring (Figures S7–S9). The sensors also exhibited high robustness in measuring the target biomarker, regardless of environmental pressure and pH levels (Figures S7–S9). The influence of temperature due to the temperature quenching effect can be corrected and compensated based on the temperature readings, which is introduced in the following sections. The selectivity of the sensors toward other ions and molecules is also tested by adding nontargeted interferences to the sensing solutions. High selectivity is observed for all three sensors, indicating their potential for target biomarker sensing in real human CSF samples (Figure 3k).

### Multiplexed Sensing and Software Design

**Figure 4:**
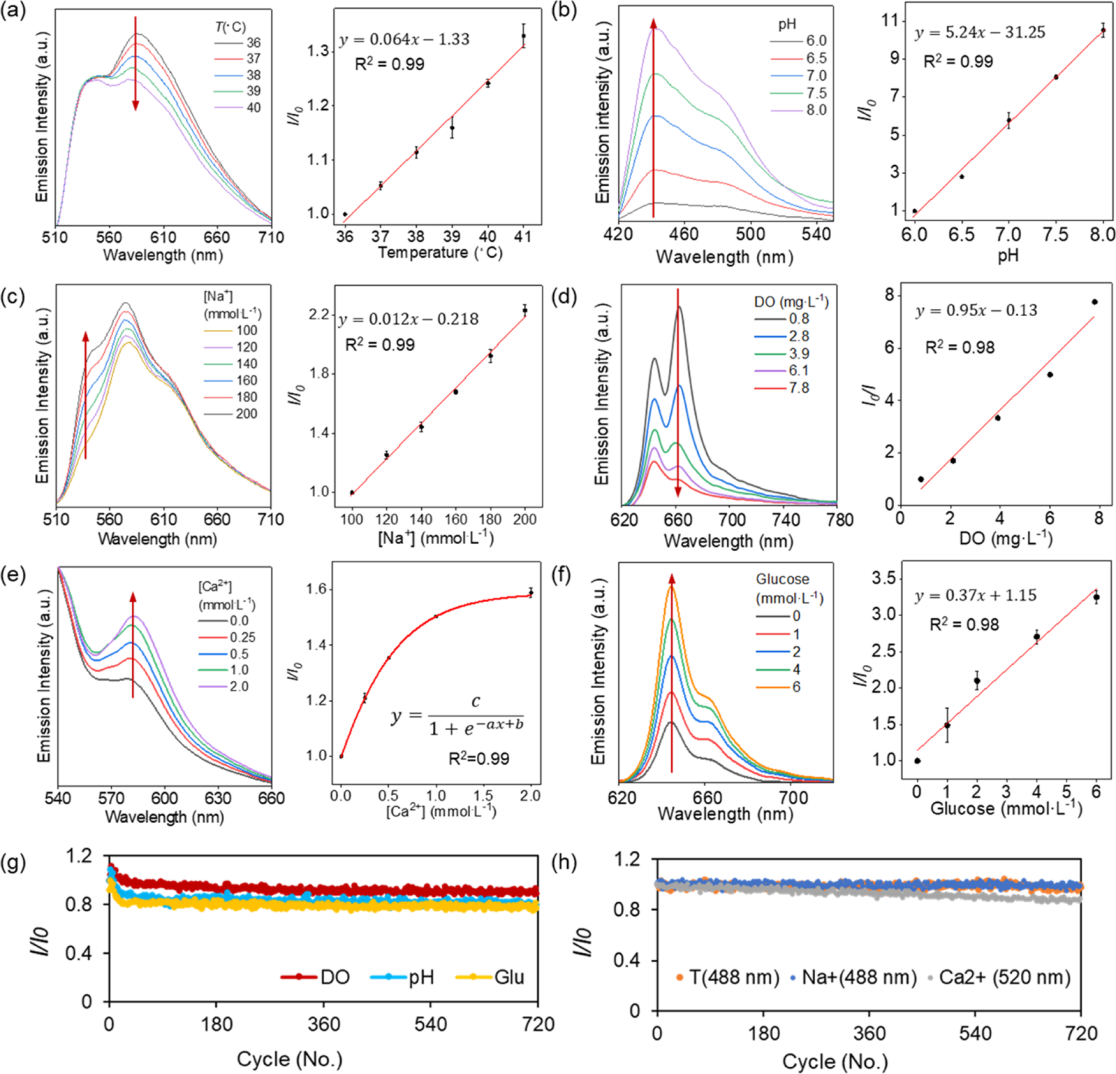
Sensor characterization using the multiplexed optical fiber bundle sensing system. Optical spectra and calibration curve of temperature (a), pH (b), Na^+^ (c), DO (d), Ca^2+^ (e), and glucose (f) sensors obtained with a series of aCSF buffer solutions under gradient target biomarker concentrations. (g) Stability test of the DO, pH, and glucose sensor under continuous pulse exposure (5s on 5s off) of 405 nm laser for 720 cycles. (h) Stability test of the temperature, Na^+^, Ca^2+^ sensor under continuous pulsed exposure of 488 nm (temperature and Na^+^), and 520 nm (Ca^2+^ sensor) laser for 720 cycles. *n* = 3.

Following the individual fabrication and characterization of the six fluorescent sensors, they were integrated onto the tips of the optical fiber bundle for multiplexed sensing (Figure 1). A multiwavelength laser, under programmed control, administered pulse excitation cycles (5s on and 5s off): a 405 nm laser for pH, DO, and glucose measurements; a 488 nm laser for Na^+^ and temperature measurements; and a 520 nm laser for Ca^2+^ measurement. In multiplexed monitoring, three lasers need to be used in turn for the measurement of the target 6 sensors. Therefore, software is designed and deployed in a microcontroller to facilitate the simultaneous measurement and concentration readout. Communications between the microcontroller, the spectrometer, and the multiwavelength laser are realized through UART. After each excitation, a spectrum is obtained using the spectrometer and saved for biomarker concentration calculation. Calibration tests of the 6 sensors in multiplexed sensing mode were performed using artificial CSF (aCSF) buffer solutions (Table S2). Similar to the characterization process for individual sensors, a series of concentrated standard buffer solutions were utilized in the multiplexed sensing characterization, each matched with corresponding laser excitations tailored to the sensors. The sensors exhibited high sensitivity toward targeted biomarkers although the emission peaks overlapped (Figure 4a–f). During the measurement, the Ca^2+^ sensor showed comparatively weaker intensity than other sensors; therefore, the spare optical fiber within the bundle was coated with a second calcium sensing film to enhance the signal. As depicted in Figure 4g,h, the introduction of pulsed laser cycles significantly improved the photostability of the sensors, marking a substantial enhancement over the prior method of continuous exposure.

As introduced in Figure 1, beyond laser and spectrometer control, the software has two additional functions, system calibration, preceding each measurement. A graphical UI is developed for user input and display via a touch screen. Prior to the initial measurement, the sensors need to be calibrated first using standard buffer solutions to ensure accurate and reliable concentration calculations. The calibration process, guided by the software’s calibration panel, involves recording corresponding spectrometer readings for subsequent correction in measurement readouts (Figure S11). For measurement readouts, computational algorithms leveraging machine learning models were designed to provide accurate calculations of the biomarker concentrations based on obtained spectra.

### Machine Learning Models for Multiplexed Monitoring

**Figure 5:**
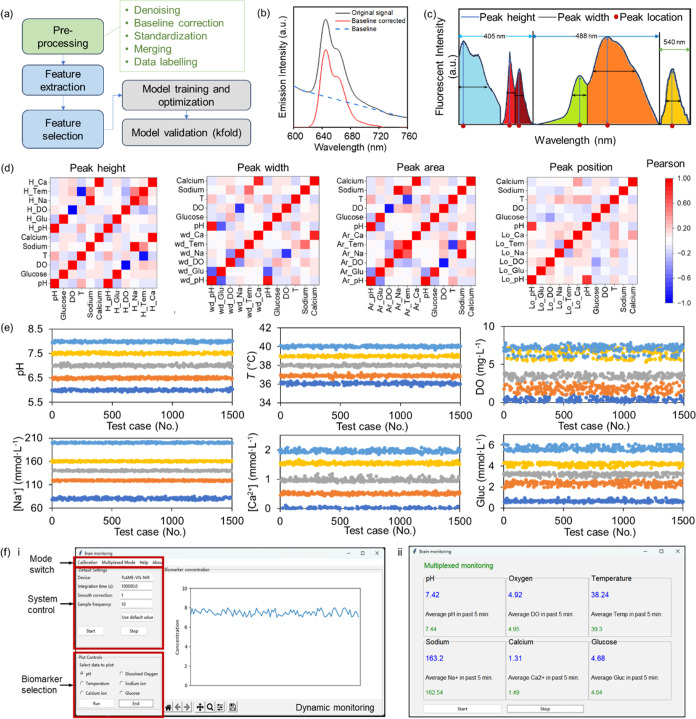
Algorithm demonstration for spectra postprocessing and biomarker concentration prediction. (a) Flowchart of the postprocessing method for the concentration prediction based on the obtained spectrum. (b) Baseline correction of the obtained optical spectrum. (c) Extracted features from the obtained spectra for machine learning model training. (d) Pearson correlation coefficient of the extracted features and the biomarker concentrations for feature selection. (e) Machine learning model prediction performance for the 6 sensors using obtained optical spectra features. (f) Designed graphical user interface for direct biomarker concentration readout. i: system control panel and dynamic readout; ii: multiplexed biomarker readout panel.

There are two major overlaps in emission spectra: (1) overlapped DO and glucose sensors’ spectra when excited by the 405 nm laser and (2) overlapped Na^+^ and temperature sensors’ spectra when excited by the 488 nm laser. Therefore, direct calculation of the concentrations based on the calibration curve would not provide an accurate readout if more than one biomarker concentration fluctuates at the same time. Computational algorithms based on machine learning were utilized to overcome the overlapping issue and provide an accurate readout. Following spectra acquisition, postprocessing algorithms were deployed and used to obtain reliable biomarker concentration readouts (Figure 5a), which includes spectrum denoising, baseline correction, standardization, spectra merging, and labeling. The spectrum was first denoised and baseline-corrected using the penalized least-squares baseline correction algorithm (Figure 5b).^37^ Each measurement provided three spectra due to the sequential use of three lasers to excite the films. These spectra were merged and appropriately labeled before their integration into multitask machine learning models. The labels for merged data sets comprise six values corresponding to the biomarker concentrations and are essential for model training.

After preprocessing, each data input contained 1380 intensity values, and several features were extracted from the data set before training (Figure 5c). Based on the sensing mechanisms and observations, the following features were extracted from the spectra data set: peak height, area under curve, prominence, peak width, and peak position. Before feature selection or model training/inferencing, the features were first corrected with exposure time for compensation of the photobleaching effect. Besides, it is believed that the model automatically learns the pH and temperature effects on the sensors from the extracted features and corresponding labels. The correlation between the extracted features and biomarker concentrations was analyzed using the Pearson correlation coefficient for feature selection (Figure 5d). The results indicated that peak height, area under the curve, and peak width showed the highest correlation to biomarker concentration, and thus were proceeded for model training.

The model training was carried out by feeding the extracted features and corresponding labels into predefined regression models. Various regression models with optimized hyperparameters were explored (Table S3) and compared based on calculation metrics such as mean square error (MSE), mean absolute error (MAE), and *R*^2^, indicating that the Bayesian model exhibited superior accuracy (Figure 5e and Table S6). The final model achieved MSE of 0.001, 0.52, 0.01, 1.31, 0.002, and 0.11; MAE of 0.03, 0.57, 0.08, 0.84, 0.04, and 0.29; *R*^2^ of 0.99, 0.93, 0.99, 0.99, 0.99, and 0.96 for the calculation of pH, DO, temperature, Na^+^, Ca^2+^, and glucose calculation, respectively (Figure 5e). Importantly, the test data set encompassed spectra from various temperatures and exposure times, highlighting the model’s efficacy in compensating for temperature variations and photobleaching.

With the successful training of the biomarker concentration measurement model, it was deployed in the microcontroller, offering direct user readout through a graphical UI (Figure 5f). The UI provided dynamic readouts, storing values of the six biomarkers in individual circular buffer vectors after each scan with three lasers and displaying the calculated concentration continuously for a maximum of 5 min (Figure 5f-left). Another frame of the UI displays the multiplexed reading of six biomarker concentrations simultaneously with an average concentration reading of the past 5 min presented to the user for references (Figure 5f-right).

### Multiplexed Sensing in an *Ex Vivo* Brain Model for Continuous Monitoring of Disease Progress

**Figure 6:**
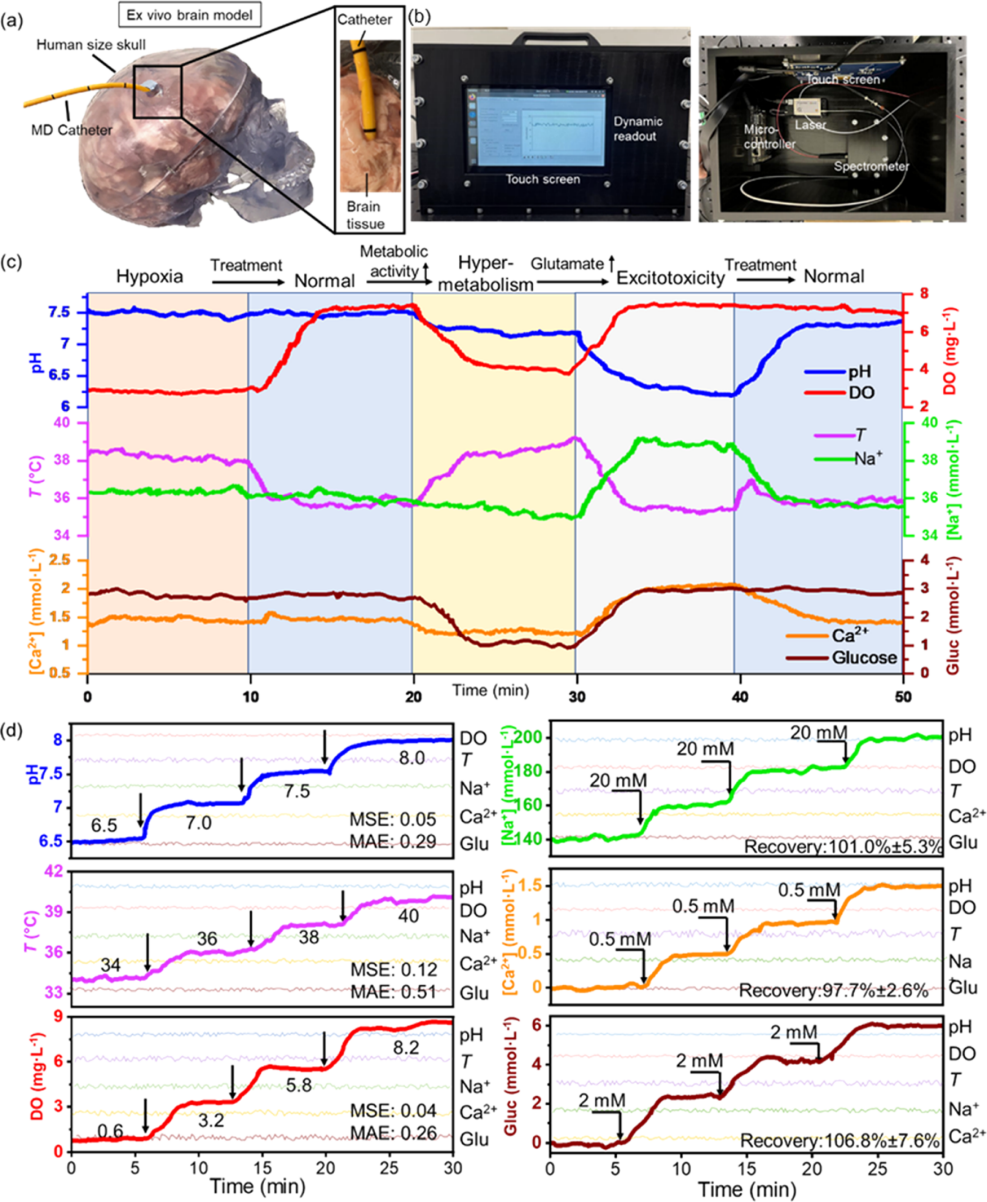
Multiplexed sensing system prototyping and validation in *ex vivo* brain models and clinical human CSF samples. (a) Photographs of the lamb brain model to perform *in situ* measurement. An optical fiber sensing system integrated in a microdialysis catheter was inserted into the brain model. Three lasers were switched to excite the sensing films. Scale bar = 50 μm. (b) Photographs of the prototyped brain monitor and the graphical user interface. (c) Continuous monitoring of three complications, hypoxia, hypermetabolism, and excitotoxicity, and recovery from the complications to healthy stages. The data shows time-dependent biomarker changes in different brain physiology stages and stage transitions. (d) Multiplexed measurement of pH, DO, temperature, and spiked Na^+^, Ca^2+^, and glucose in pooled human CSF samples, *n* = 3. mM: mmol L^–1^.

Secondary injury is a common complication in TBI, occurring gradually after the initial brain insult due to brain inflammation, elevated pressure, swelling, etc. It plays a major role in long-term brain damage and death. Unlike the first insult to the brain, secondary brain injury can be partly prevented through close monitoring of the brain physiologies to identify complications and take immediate interventions.^38^ Herein, we assess the developed system for multiplexed continuous monitoring of brain physiology progression after TBI using lamb brain models (Figure 6a). Fluorescent sensors for the six biomarkers were coated on the fiber bundle tips (Figure S12) and inserted into a CSF drainage catheter, facilitating interactions between the sensing films and brain biomarkers in deep brain tissues. Multiwavelength laser was used controlled with the designed program for automatic excitation of the fluorescent sensing films, and real-time fluorescence signals were collected by the spectrometer and analyzed by postprocessing algorithms. The whole sensing system was prototyped and assembled with a touch screen on a readout plastic box fabricated by using 1 cm acetal sheets (Figure 6b). Artificial CSF (aCSF) buffer solutions were prepared according to references to simulate the brain physiologies after TBI. For the measurement, clean lamb brains were immersed in these prepared solutions with the designed system catheter inserted inside, and the brain phase transitions were established by continuous extraction and addition of the prepared aCSF solutions. Through continuous monitoring of the *ex vivo* TBI brain model using the designed fiber bundle system, after the initial insult, brain hyperthermia and hypoxia were observed as identified by a high temperature of 38 °C and low DO of 3.2 mg L^–1^, which can be triggered by acute phase response, brain inflammation, and reduced oxygen supply in clinical cases (Figure 6c).^39^ By alleviating brain pressures, the blood flow rate can be increased, resulting in the amelioration of hypoxia (Figure 6c). As brain damage progresses, TBI-induced mitochondrial dysfunction may lead to decreased respiratory rates, reduced ATP production, and depletion of glucose and oxygen, causing another brain complication called hypermetabolism. As shown by the system readout, elevated temperature (39 °C), decreased DO (3.8 mg L^–1^), and glucose (1 mmolL^–1^) were observed in this stage.^39^ The cascading of mismatched processes of metabolism and cerebral overflow with accumulated sodium and calcium ions creates excitotoxicity as indicated by decreased pH (6.5) and increased Na^+^(180 mmolL^–1^) and Ca^2+^ ions (3 mmolL^–1^).^40^ After proper medication to stabilize the mitochondrial membrane, excitotoxicity can be treated to a normal stage (Figure 6c). The system’s prediction accuracy was evaluated using MSE and MAE of the predicted value with the prepared buffer concentrations (Table S4). An MSE of 0.89, 0.07, 0.01, 22.54, 0.04, 0.13, and an MAE of 0.76, 0.21, 0.26, 3.83, 0.50, and 0.9 were obtained for temperature, DO, pH, Na^+^ ions, Ca^2+^ ions, and glucose sensors, respectively, indicating promising results for continuous and precise biomarker monitoring. The designed system offers long-term monitoring of 6 biomarkers simultaneously with high accuracy and the system has a fast response (<5 min) for the tracking of disease progress. Both increasing and decreasing biomarker concentrations were correctly identified in three disease complication models, suggesting potential for clinical brain CSF monitoring and timely treatment of secondary brain injuries.

### System Validation Using Clinical Human CSF Samples

Clinical human CSF sample was obtained and used for further validation of the multiplexed sensing system in clinical applications. Human CSF samples were collected from 11 patients admitted to St Mary’s Hospital, London. Multiplexed measurement of 6 biomarkers that were spiked in the collected human CSF was performed (Supporting Information 8) in continuous mode to assess the system ability to track the accumulation of brain biomarkers with the presence of diverse brain molecules. Before measurement, the sensing system was first calibrated using two prepared aCSF buffer solutions that contained the target biomarkers at specific concentrations (Table S5, Supporting Information). The acquired spectra during calibration were subsequently used to ensure an accurate measurement of the human CSF samples. As shown in Figure 6d, the designed optical fiber bundles sensing system successfully measured a series of increasing pH, temperature, and DO values in the CSF sample. The system readout demonstrated high accuracy in quantifying pH, temperature, and DO with MSE values of 0.05, 0.12, and 0.04, and MAE values of 0.29, 0.51, and 0.26, respectively. Due to the unknown original concentrations of [Na^+^], [Ca^2+^], and glucose in the sample, the system’s ability in recovering the biomarker’s variations was evaluated by calculating the system’s recovery rate following the addition of the biomarkers. Despite the existence of numerous nontarget molecules in the human CSF sample, the system demonstrated a good recovery rate of 101 ± 5.3, 97.7 ± 2.6, and 106.8 ± 7.6% for the measurement of increased [Na^+^], [Ca^2+^], and glucose, respectively. The results indicated the system’s proficiency in precisely revealing elevated ions and metabolites, enabling accurate brain CSF monitoring of patients experiencing brain hyperthermia, excitotoxicity, and hypometabolism.^40^ As indicated by the continuous readout of the CSF samples, the system exhibited a high stability in long-term measurement after compensation for environmental variations and photobleaching. These findings further imply that the sensing system can selectively and accurately identify the target biomarkers, offering good precision and robustness in real clinical applications.

## Conclusions

A miniaturized and multiplexed optical sensing system was developed using self-designed optical fiber bundles and fluorescent sensors for simultaneous measurement of 6 biomarkers in brain CSF. Machine learning-based computational postprocessing algorithms were integrated to provide dynamic and multiplexed biomarker monitoring with high accuracy and robustness in various environmental conditions, showing a promising potential for clinical brain CSF monitoring. In the first part, a fiber bundle was designed and fabricated for multiplexed sensing with a specific focus on maximizing emission collection and light transmission. Six fluorescent sensing films were successfully optimized and integrated with the designed bundle for temperature, DO, pH, Na^+^, Ca^2+^, and glucose measurement with desired sensing performance. The fluorescent indicators and encapsulation polymeric supports were optimized according to the properties of the target analytes to provide high sensitivity, selectivity, stability, and reversibility. In the current design, the system is developed for the monitoring of 6 biomarkers simultaneously with one spare fiber to accommodate a seventh biomarker. However, it is not the maximum capacity of the system. With careful optimization of the connector and individual fiber dimensions, it has the potential to measure more than 10 biomarkers concurrently. Achieving this would require additional lasers with varying wavelengths to efficiently excite all of the sensors. New probes would need distinct excitation or emission wavelengths to minimize spectral overlap. Recent advancements in narrow-band fluorescent probes engineered indicate that simultaneous measurement of more than 10 biomarkers is feasible.^41^ In the future, with the development of appropriate recognition elements, this optical fiber bundle system could be adapted for the detection of more complex biomarkers, including proteins such as S100b, interleukin 6, and tau, enabling more comprehensive brain monitoring. During actual measurement, a trade-off exists between fiber sizes and signal intensity as larger fiber cores provide stronger signals but come with increased probe dimensions. In the future, tapered or D-shape fibers with larger sensing areas could be employed to enhance signals without compromising overall sizes.^42,43^

The system was developed by using soft, safe, and highly biocompatible materials, along with probe miniaturization, to minimize potential damage or inflammation from long-term implantation. The probes are physically entrapped in polymer matrices at the distal end of the fiber with optimized pore sizes to minimize leaching (Figure S13). During measurements, CSF is collected in a drainage catheter and analyzed by the sensors. This catheter ensures sufficient CSF collection, maintains a relatively stable background noise level, and prevents probe leaching into the brain tissue. Given the low toxicity, minimal leaching, and small volume of each probe, the system is expected to pose minimal risk and cause negligible damage during continuous long-term monitoring. However, the biocompatibility of the system needs to be thoroughly evaluated through comprehensive studies to assess the cellular and tissue responses over extended periods. In the future, hydrogel or silk fibers can be leveraged to further improve the biocompatibility of the sensing system.^11^ Inflammation sensors including Interleukin 6 and Interleukin 10 sensors can also be integrated to the sensing bundle by designing proper recognition probe with fluorescent labels to monitor both TBI-related complications and long-term implantation induced infections to prevent adverse outcomes.^44^

In the second part, a software system was developed for the automated multiplexed measurement of these six biomarkers concurrently. The software controls the laser and spectrometer based on user preferences and offers accurate and continuous readout of 6 biomarkers’ concentrations simultaneously, relying on the embedded pretrained machine learning model. To achieve fully automated monitoring, a multiwavelength laser containing three lasers was utilized and controlled by the microcontroller for pulsed laser output and synchronized spectrum readings. A pretrained model was built with the capability to provide a highly accurate calculation of biomarker concentrations based on overlapped fluorescent spectra under various environmental conditions and exposure times. Additionally, a graphical user-friendly UI was deployed in the microcontroller for direct user interaction. In the validation tests, the optical fiber bundle sensing system successfully tracked concentration variations in three TBI brain models. Sensitively and accurate capture of brain deterioration are critical for medical devices. The current study achieved promising results using shallow machine learning models based on feature engineering. However, there is room for improvement in accuracy in future studies. With advancements in AI, deep learning architectures such as CNN, recurrent neural networks, and transformer models can be explored to extract deeper features that account for time-dependent correlations in biomarkers, potentially enhancing the precision and accuracy of the sensors.^45^ The control software and UI design may benefit from further refinement based on feedback from clinical physicians to improve usability and integration with other devices in ICU settings.

In the measurement of clinical human CSF samples, the multiplexed sensing system demonstrated high sensing precision for the continuous measurement of multiple biomarkers simultaneously with high selectivity and stability. Therefore, we conclude that the sensing system, coupled with intelligent algorithms, possesses great potential in the multiplexed monitoring of deep brain biomarkers for TBI treatment. The device also holds potential to be utilized with other modalities. The device’s optical fiber-based design offers full compatibility with magnetic resonance imaging (MRI), making it suitable for use in MR-guided surgeries with long-distance fibers and remote monitoring capabilities. It could also be integrated with drug delivery systems, utilizing hollow fibers to directly measure responses to pharmacological interventions.^46^ Further studies are necessary to assess the performance of the sensing system in diverse environments to fully explore its potential in multiplexed sensing and beyond.

The system is minimally invasive, multiplexed, sensitive, selective, robust, and fully reversible and suitable for long-term applications. It holds great promise for precise and continuous monitoring of deep brain physiology, aiding in pathological identification and clinical guidance in various clinical cases, including but not limited to brain injury, ischemic or hemorrhagic stroke, and brain tumor resection.^47,48^

## Experimental Section/Methods

### Fabrication of Optical Fiber Sensors

#### Temperature Sensor

The temperature sensor was fabricated by encapsulating Ru(bpy)_3_ in epoxy polymer. To fabricate it, 0.5 mL of 1 mmol·L^–1^ Ru(bpy)_3_ was first prepared by dissolving 0.5 mmol Ru(bpy)_3_ in 0.5 mL of pure ethanol and thoroughly mixed with the same volume of epoxy resin. Then, the mixture was placed on the hot plate at 80 °C for 1 h to remove the ethanol. After that, the epoxy hardener was added to the above mixture in the ratio of 1 (hardener) to 2 (epoxy resin). Dip coating (1 cm s^–1^) was then performed to coat the film on the tip of the fiber coupler and the film was cured at room temperature for 72 h. The fiber sensor was washed with pure deionized (DI) water for several minutes before use.

#### DO Sensor

For the fabrication of PDMS-PdOEP sensing film, 20 mg of prepolymer (Sylgard 184) was mixed with 2 mg of curing agent with a spatula. The mixture was fully mixed until we got a homogeneous and transparent solution. The indicator PdOEP was dissolved in THF with a concentration of 1 mmol L^–1^ for a total volume of 500 μL. The PDMS mixture was then added to the PdOEP/THF solution and stirred for several minutes until fully mixed. Gas was removed from the mixture by using a vacuum. Dip coating was then performed with the prepared solution. The film was left to dry and cure at 80 °C for 3 h.

#### pH Sensor

One mg portion of HCC was dissolved in 1 mL of ethanol. Then, 50 μL tetraethyl orthosilicate (TEOS), 100 μL HCC solution, 20 μL 0.1 mol L^–1^ HCl, and 10 μL DI water were mixed and stirred thoroughly for 1 h to obtain homogenized sol–gel solutions. The mixture was subsequently aged at room temperature for 1 h before coating. Dip coating was performed and left for 3 days for fully gelation.

#### Sodium Sensor

To fabricate the PEGDA film, 156.5 μL of PEGDA and 10.6 mg of AM were mixed with 2-HMPP (2%, v/v) in DI water with a ratio of 1:2 (v/v). Stock solution (1 mmol·L^–1^ CoroNa Green in DMSO) was also added with the amount to result in the optimized probe concentration (Figure S3, Supporting Information). The solution was then dip-coated onto the fiber tip and placed under UV light for 3 min.

#### Calcium Sensor

The polymerization solution consisted of 350 mg of acrylamide (35.0% w/v), 20 μL of dimethyl sulfoxide (DMSO) dissolved (20% w/v) *N*,*N*-methylenebis(acrylamide), and 980 μL of DI water. Fluorescent probe Rhod-5N stock solution was then added to the polymerization solution to obtain 50 μmol L^–1^ indicator concentration. The polymerization was initiated with 24 μL of a 10% ammonium persulfate solution and 12 μL of *N*,*N*,*N*′,*N*′-tetramethylethylenediamine (TEMED), and the solution was stirred at room temperature until well mixed. Dip coating was performed and was left for polymerization in fume hood for 1 h.

#### Glucose Sensor

The glucose sensor structure has two layers. The top layer is a polyacrylamide-based glucose oxidase layer for glucose oxidation. The bottom layer is a sol–gel-based oxygen sensing layer and was coated on the fiber tip. The sol–gel-based oxygen sensing layer was fabricated by mixing 0.46 mL TEOS, 0.04 mL GLYMO, 0.6 mL ethanol, 0.2 mL DI, 8 mg PtOEP, and 0.2 mL of HCl (0.1 mol L^–1^) together and stirring thoroughly for 1 h to obtain homogenized sol–gel coating solutions. The sol–gel solution was then dip-coated to the fiber tip and aged under room temperature to form a clear and homogeneous sol–gel oxygen sensing film. The top layer coating solution was prepared by mixing 50 μL 4 mmol L^–1^ glucose oxidase PBS solution with hydrogel precursor solution, prepared by 37.7% (w/v) acrylamide, 15 μL DMSO dissolved (20% w/v) *N*,*N*-methylenebis(acrylamide), and 2 μL 2-HMPP in DI water. After the top layer was coated on the first layer, the film was polymerized under UV light for 3 min.

### Fiber Bundle Fabrication

The bundle comprises 6 optical fiber sensors and 1 extension optical fiber (silica, multimode fiber, core/cladding: 105/125 μm) that are bundled together and evenly distributed in a subminiature version A (SMA) connector (380 μm bore size) to ensure equivalent light transmission. The fabrication process of the bundle included 5 steps: fiber tip stripping; fiber fixing into a connector; fiber tip cutting; fiber polishing; and fiber securing (Figure S1, details in the Supporting Information).

### Design and Development of the Fully Automated Optical Sensing System for Multiplexed Monitoring

#### System Hardware Design

In order to achieve fully automated measurement of the biomarkers, a multiwavelength laser that consists of three lasers (405, 488, and 520 nm) was used here instead of using three collimated individual lasers that requires manually switch. Therefore, the fiber bundle sensing system consists of a multiwavelength laser light for fluorescent sensing film excitation, a spectrometer for fluorescent spectra capturing, a self-designed fluorescent sensing film integrated optical fiber bundle for 6 brain biomarker measurements, a fiber coupler for the connection of the optical equipment, a microcontroller (NVDIA Jetson Developer Kit) for laser/spectrometer control, biomarker concentration calculation, and UI readout, and a touch screen (Waveshare) for user control and data display.

#### Multiplexed Sensing System Characterization

The new system was recalibrated by using artificial CSF buffer solutions for multiplexed monitoring. The following buffer solutions were utilized for the calibration of the sensors, one after another. For the procedure, the optical fiber bundled sensors were inserted into the solution together and the corresponding laser (for pH, DO, and glucose; 405 nm, for Na, temperature sensor: 488 nm, and for Ca^2+^ sensor: 520 nm) was lit on controlled by the controller. The sensors’ fluorescent intensities and spectra were measured with the spectrometer and saved in the controller for offline analysis. Details are given in Supporting Information.

### Signal Post Processing and Machine Learning Modeling for Multiplexed Biomarker Readout

After the multiplexed spectra are obtained, the signals are smoothed, corrected for baseline drifts, and standardized before feature extraction and model training (Supporting Information).

#### Feature Extraction

The following features were extracted for machine learning model trainings with the aim of building a robust intelligent model for accurate biomarker concentration prediction.

##### Peak Height

The key feature is the peak height because it changes significantly with different values of biomarkers due to the fluorescent sensing principles. There are six major peaks in the spectrum, and they are taken as the intensity values of six fixed wavelengths. The peaks at 34, 28, 341, 725, 884, and 1262 are correlated with the pH, glucose, DO, Na^+^, temperature, and Ca^2+^ levels of the sample, respectively.

##### Peak Width

Another feature is the peak width, which changes in accordance with the corresponding biomarker concentrations. Although the changes might not be as significant as peak height, they were still extracted from the spectra as an input for model training.

##### Area under Curve

The area of each peak was also used as another feature for model training. It was calculated based on the trapezoidal integration rule. The boundaries of each peak are defined by finding the local minimum values.

##### Peak Position

It is noticed in the spectra that the peak shifts slightly at different concentrations of biomarkers. Therefore, the peak position is computed as another feature for training the models. The peaks are located by finding the local maximum.

Before the features were fed into the model, the features were first corrected with exposure time to compensate for the photobleaching.

#### Feature Correction with Exposure Time for Photobleaching Compensation

The extracted features need to be corrected for photobleaching, which corresponds to the peak features extracted at time 0. Therefore, the peak height, width, and area correction with exposure time are modeled here.

The photobleaching can be modeled using an exponential equation, eq 1.
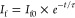
1where, *I*_f_ denotes the emission intensity at the moment of measurement, *I*_f0_ is the initial emission intensity at *t* = 0, *t* is the exposure time, and τ is the photobleaching time constant index.

Therefore, the intensity when the calibration (*t* = 0) is performed can be calculated as
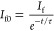
2

Therefore, for the peak intensity at time 0, it should be *I*_f0_. Here, we assume the fluorescent peaks follow the distribution of the Lorentzian curve, and thus the peak width remains the same. Peak area can be estimated as
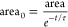
3Using the photostability data obtained for each sensor, we were able to calculate the photobleaching constant τ for each sensor using least-squares fitting methods and they were used for the photobleaching compensation.

The constants τ for temperature, DO, pH, Na^+^, Ca^2+^, and glucose are 51.13 (*R*^2^ = 1), 48.8 (*R*^2^ = 0.97), 37.0 (*R*^2^ = 1), 22.0 (*R*^2^ = 0.99), 38.0 (*R*^2^ = 0.99), 133.7 (*R*^2^ = 0.96), respectively.

#### Feature Selection Using Pearson Correlation Coefficient

Pearson Correlation was calculated to study the correlation between the features and the biomarker concentrations. Only strongly correlated features were included for the model training.

#### Model Training

Various machine learning regression models were developed and optimized to achieve optimal performance. The models are Linear regression model, Ridge regression model, Lasso regression model, and Bayesian regression model (Supporting Information).

#### Model Validation

The complete data set underwent a 75/25 split for training and testing purposes, with 75% allocated to training and 25% to testing. Parameter tuning and model comparison were conducted on the training data set using a 10-fold cross-validation approach. This entailed partitioning the training set into 10 subsets, with 9 used for training and 1 for validation, iterated 10 times. Validation accuracy was assessed using metrics such as *R*^2^, MSA, and MSE. Through this iterative validation process, model hyperparameters were optimized, and the most effective model was selected by comparing the outcomes of various regression models. Following the identification of the optimal model, it underwent testing using the reserved testing data set, which had not been previously utilized for model training. Performance evaluation was based on metrics, including MSE, MAE, and *R*^2^.

### Readout Box Design and Assembly

#### UI Design

A user-interface is developed using Tkinter in python for user control of the system and a continuous biomarker concentration continuous readout. It includes four parts. The first part is system control, where the users can input the desired readout options, including the temporal resolution, single biomarker continuous temporal readout, or multiplexed 6 biomarker readout. The parameters for the spectrometer reading, including integration time and smooth level, can also be determined. The control of the lasers and spectrometer is realized via a USB communication protocol by using a serial library in python.

The second part is the calibration module. In this module, the fabricated multiplexed bundle is instructed to be placed in standard buffers (Table S4). Once immersed in the buffer, the software would control the laser lights to shine the three wavelengths one by one, and the corresponding spectra would be recorded in the system. Then, the users would be instructed to switch to the second calibration buffer, and once the button is clicked, the lasers would be lit on one after another with spectra taken and saved in the controller memory for continuous readout correction.

The third part is a multiplexed biomarker readout. In this module, the software allows the user to visualize the concentration of 6 biomarkers simultaneously. In this mode, the laser lights are turned on one by one, and the spectra are recorded and fed into the machine learning model described in Signal Post Processing and Machine Learning Modeling for Multiplexed Biomarker Readout Section. The calculated biomarker concentrations are stored in 6 different circular buffers (with size for 100 data storage). The average value of the past 5 min (34 most recent data points) is calculated and displayed on the UI.

The fourth part is the single biomarker temporal readout. In this module, the users can select the target biomarker that they are interested to monitor. Based on their selection, the time series of the biomarker concentration value stored in its circular storage buffer is plotted on the software UI for user visualization.

#### System Fabrication and Assembly

The readout box is designed using Solidworks. It is fabricated using 1 cm thick black acetal plastic sheets. The readout box includes a spectrometer, a multiwavelength laser system, an edge computer (Nvidia, Jetson Xavier), a touch screen for user-interface, and a fiber coupler. The edge computer is utilized for signal processing, laser and spectrometer control, and result readouts. The fiber coupler is used for light transmission between the laser system, designed optical bundle sensor, and the spectrometer. All components were fixed by using screws. The readout box is miniaturized, compact, and lightweight.

### System Validation Using the *Ex Vivo* Brain Model

Three TBI complications were simulated using lamb brain models to test the proposed system’s ability in identifying secondary brain injuries in TBI patients. The three complications or secondary injury scenarios are hypoxia, hypermetabolism, and excitotoxicity. After the initial insult, the brain usually experiences a high temperature and oxygen deficiency, leading to brain hypoxia. In the hypermetabolism state, apart from the symptoms in hypoxia, the brain also has lower energy supply shown by lower glucose levels due to excess metabolic activities. Whereas in excitotoxicity, the brain usually suffers from more acidic environments and elevated sodium and calcium ion levels. The buffer solutions for these three scenarios are listed in Table S5. Before measurement, the sensors were first calibrated following the instruction of the calibration panel of the software, and the calibration spectra were saved for accurate real-time continuous measurement. The prediction output from the sensing system on the biomarker concentrations were compared with the corrected biomarker concentrations in sample preparation using mean squared error (MSE) and mean absolute error (MAE). The equations are as follows:
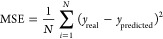
4
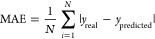
5

### Human CSF Measurements

This study received approval from the hospital research governance team at St Mary’s Hospital, London, U.K. (15SM3116), and the London Stanmore Research Ethics Committee (16/LO/0183). All patients gave written informed consent before taking part in the study, which was given by the author (MF). The study conformed to the precepts set out in the Declaration of Helsinki of 1975.

Human CSF were collected from 11 patients admitted to St Mary’s Hospital, London, U.K.

Specimen collection details can be found in ref (49). In short, a spinal catheter was inserted into the patient’s lumbar by an anesthetist, and 5 mL of CSF was collected into a polypropylene tub. Once taken, samples were centrifuged at 3000*g* for 10 min at room temperature and stored at −80 °C until analysis.

Before analysis, samples were diluted with aCSF to obtain 60 mL of CSF and the measurement details are described in Supporting Information, Section S7.

## Supporting Information Available

Detailed experimental methods and characterization results for the multiplexed sensing system. Signal processing and modeling methods and results are also included (PDF)

## Notes

**Ethics Approval Statement** This study received approval from the hospital research governance team at St Mary’s Hospital, London, U.K. (15SM3116), and the London Stanmore Research Ethics Committee (16/LO/0183). All patients gave written, informed consent before taking part in the study, which was taken by the same author (M.F.). The study conformed to the precepts set out in the Declaration of Helsinki of 1975.
