# Supplementary material for: Fully Automated and AI-Assisted Optical Fiber Sensing System for Multiplexed and Continuous Brain Monitoring
Source: ACS Sens. 2024 Dec 4;9(12):6605–20. doi: 10.1021/acssensors.4c02126 (PMC11686509; doi:10.1021/acssensors.4c02126)
Supplement: Supplementary file 1 — se4c02126_si_001.pdf [file se4c02126_si_001.pdf]

Supporting Information

A Fully Automated and AI-assisted Optical Fiber Sensing System for Multiplexed and Continuous Brain Monitoring

Yuqian Zhang[a], Naihan Zhang[a,b], Yubing Hu[a], Christopher Pereira[c], Michael Fertleman[c], Nan Jiang[d,e], Ali K. Yetisen[a,]*

Table of Contents

Methods 2

1. Materials and equipment 2
2. Bundle fabrication and film coating method optimization: 3
3. Sensor characterization 4
4. Multiplexed sensor characterization 10
5. Signal post processing and machine learning modeling of the obtained spectra 11
6. System validation using ex vivo brain model 14
7. Human CSF measurements 15

Results 17

Methods

1. Materials and equipment

Materials: Tris(2,2'-bipyridyl)dichlororuthenium(II) hexahydrate ($Ru(bpy)_3$, 99.95%), 1,4-Bis(2,3-epoxypropoxy)butane, 2,2'-[(1-methylethylidene)bis(4,1-phenyleneoxymethylene)]bisoxirane, 7-Hydroxycoumarin-3-carboxylic acid (HCC, 98%), 2,3,7,8,12,13,17,18-Octaethyl-21H,23H-porphine palladium(II) (PdOEP, 85%), Tetraethyl orthosilicate (TEOS, 98%), (3-Glycidyloxypropyl)trimethoxysilane (GLYMO, 98%), 3-(trimethoxysilyl)propylmethacrylate, Dimethyl sulfoxide (DMSO, 99%), Acetone (99.9%), Poly(ethylene glycol) diacrylate (PEGDA Mn700), Tetrahydrofuran (THF, 99%), Acrylamide, N,N'-Methylenebisacrylamide (MBA), Glucose Oxidase, Platinum octaethylporphyrin (PtOEP, 95%), Tris base (99%), Tris hydrochloride (99%), Phosphate buffered saline, sodium chloride (NaCl) (99.5%), calcium chloride ($CaCl_2$) (93%), potassium chloride (KCl) (99%), magnesium chloride ($MgCl_2$) (98%), zinc chloride ($ZnCl_2$) (98%), sodium phosphate dibasic (Na_2HPO_4) (99%), sodium phosphate monobasic (NaH_2PO_4) (99%), copper(II) chloride (93%), D-(+)-glucose (99.5%), L-ascorbic acid (99%), uric acid (99%), sodium nitrite ($NaNO_2$) (97%), albumin from porcine serum (98%), hydrochloric acid (HCl) (37%), nitric acid (HNO_3) (70%), sodium hydroxide (NaOH) (97%), 5-hydroxyindole-3-acetic acid (98%), and ethanol (99%) were purchased from Sigma-Aldrich. SYLGARD™ 184 Silicone Elastomer was purchased from Dow. Sodium L-lactate (98%), sodium pyruvate (99%), and dopamine hydrochloride (99%) were purchased from Alfa Aesar. CoroNa Green and Rhod-5n were purchased from Invitrogen™. All chemicals were reagent grade and used as received without further purification.

Equipment: For the fiber bundle fabrication, the following items were purchased from Thorlabs: LF5P-5 µm Grit Silicon Carbide Lapping (Polishing) Sheets, LF3P-3 µm Grit Aluminum Oxide Lapping (Polishing) Sheets, LF1P-1 µm Grit Aluminum Oxide Lapping (Polishing) Sheets, D50-SMA - SMA Connector Polishing Dis, FTS4- Three-Hole Stripping Tool, S90R- Ruby DualScribe Fiber Optic Scribe, FG105UCA-Multimode Fiber, Ø105 µm Core, 10380A-Standard SMA905 Multimode Connector, Ø380 µm Bore, and TT400R5S1B - 1x2 Multimode Fiber Optic Coupler, Low OH, Ø400 µm Core, 0.39 NA, 50:50 Split. FLAME-S-VIS-NIR-ES spectrometer assembly (350–1,000 nm) (Ocean Insight) was used to acquire the fluorescent spectrum. Collimated laser diode modules including 405nm, 4.5mW (CPS405), 450 nm, 4.5 mW (CPS450), and 520 nm (4.5 mW) diodes were purchased from Thorlabs for film excitation. Microplate reader (Thermo Scientific Skanlt) was also used for

sensing probe characterization. The micrographs were obtained by an upright microscope for polarization (DM2700 P; Leica). A hotplate stirrer (Fisherbrand Isotemp) was used to homogenize the sol-gel precursor solutions and control the temperature of the buffer solutions during experiments. A pH meter (FiveEasy F20; Mettler Toledo) was used to measure the pH value. An oxygen benchtop meter (HI-2004 Edge; Hanna Instruments) was used as a golden standard to prepare DO buffer solutions. The obtained spectra obtained during the experiment were analyzed offline using Python code. Ocean View 2.0 and Thermo Scientific Skanlt were used as the software to analyze data from the spectrometer and the microplate reader, respectively. iPhone 11 camera was used to capture the photographs.

2. Bundle fabrication and film coating method optimization:

2.1 Fiber bundle fabrication

The fabrication process involved the following steps (**Figure S1**): 1. Optical fibers were stripped to a length of 1 mm and cleaned with ethanol. The fiber connector was soaked in ethanol and placed on a clean, dry surface for cleaning. 2. Epoxy was injected into the connector using a syringe until a small bead appeared on the connector ferrule's outer face. 3. Seven prepared optical fibers were inserted into the connector, and epoxy was applied at the contact area between the connector end and a furcation tubing. The epoxy was left to dry overnight for stability and the fibers were cut using a fiber scribe, ensuring the fiber length above the epoxy was less than 1 mm. 4. The polishing disc and connector were cleaned, and the connector was inserted into the polishing disc. Fiber tips were polished using 1 μm, 3 μm, and 5 μm aluminum oxide polishing film, with gradual pressure increase to maintain light contact between fibers and film. 5. A strain relief boot was placed on the connector for protection, and epoxy was applied to secure the boot. Finally, the polished fiber end faces were inspected using a microscope for preliminary quality assessment.
The fiber tips were salinized before sensing film coating. The fiber tips were salinized in a solution of 3-(trimethoxysilyl)propylmethacrylate in acetone at a ratio of 1:50 (v/v) overnight. Once salinization is completed, the fibers were wiped clean and stored in dark.

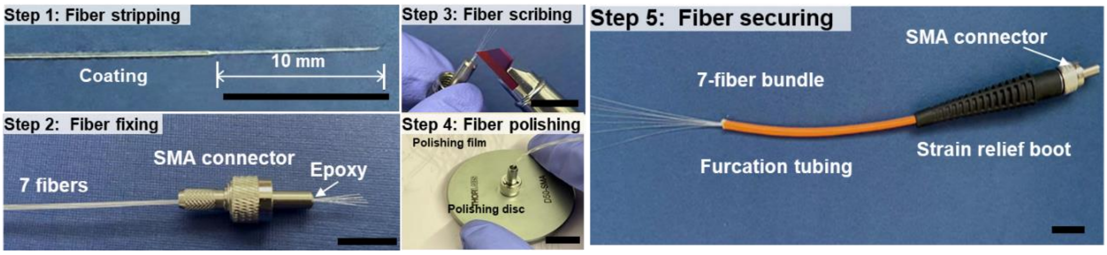

Figure S1. Bundle fabrication and test. (a) The fabrication process of the fiber bundle in 5

steps: fiber stripping, fiber fixing, fiber scribing, fiber polishing, and fiber securing. Scale bar: 1 cm.

2.2 Film coating methods:

Before fabricating sensing films on the fiber tips, coating methods were optimized to obtain the optimal fluorescent signals. The experiment was conducted using sol-gel fluorescent pre-gel solutions and different dip coating methods were used for film coating.

Sol-gel precursor solutions were prepared by mixing 0.46 mL TEOS, 0.04 mL GLYMO, 0.6 mL ethanol, 0.2 mL DI, 8 mg dye or probe, and 0.2 mL of HCl (0.1 mol L^{-1}) and were stirred thoroughly for 1 hour to obtain homogenized sol-gel coating solutions. The mixture was subsequently aged at room temperature for 1 h before coating. Coating optimization was conducted using one 60 μm core pigtail fiber and the fiber tips were stripped, cleaved, and salinized before coating.

Optimization 1: Dip coating speed

To achieve precisely controlled speed, a pump was used to facilitate the dip coating process. One side of the fiber was tied to the pump and the speed was controlled using the pump. 0.1 cm/s, 0.5 cm/s, 1 cm/s, 5 cm/s, 10 cm/s coating speeds were used for dip coating. After coating, the film was air dried for 1 hour before signal measurement. Transmission of the film (optical density) was analyzed using a spectrometer to determine the best coating speed.

Optimization 2: Layer numbers

Layer numbers are directly related to coating thickness. The smallest coating speed was used for the dip coating of each layer. The layers were dried in air for 1 h before coating the next layer. 1-5 layers were coated and studied to find the correlation between the film intensity and layer numbers. Transmission of the film (optical density) was analyzed using a spectrometer to determine the coating layers.

3. Sensor characterization

3.1 Temperature sensor

Calibration: The temperature sensor was characterized using 50 mL of pH 7.4 PBS solutions, which was was heated incrementally from 33 to 42 °C. The sensor was placed in the solution and excited with a 450 nm laser light source through a fiber coupler. The spectrum at each temperature was recorded, and the correlation between solution temperature and 600 nm intensity was analysed.

Reversibility: The temperature sensor was cycled between a 36 ℃ PBS solution and a 40 ℃ PBS solution with the same pH for 10 repetitions, recording the intensity at 600 nm each time.
Photostability: The sensor was excited continuously for 3 hours in a 36 ℃ PBS solution, and the excitation intensity at 600 nm was continuously recorded for stability test.
pH dependence: The pH dependence test was performed by immersing the sensor in in pH-varied (6.0, 6.4, 6.8, 7.2, 7.6, 8.0) 36 ℃ PBS solutions, and the emission intensity at 600 nm wavelength was analysed for its responses to different pH values.
Pressure dependence: The optical fiber sensor was submerged to the buffer solution at different depths (0–30 cm) to simulate human physiological pressure ranges according to the equation:

$$Pressure = \rho * g * h \quad \text{(Eq. S1)}$$

, where ρ refers to the buffer solution's density (approximately 1 g/cm^3), g is gravity, and h represents depth. Correlation between dipping depths and 600 nm emission intensity was analyzed.
Selectivity: Sensor selectivity was tested for cross-sensitivity to major brain CSF biomarkers. PBS solutions containing major interferent biomarkers were prepared at specific concentrations (Table S1). The sensor was then dipped into these solutions and the intensity at 600 nm was recorded for further analysis.

Table S1. Composition of interference solutions

Interference solutions	Compounds added	Concentration in cerebrospinal fluid	Concentration in interference solutions
Na^+	NaCl	110-170 mmol L^{-1}	170 mmol L^{-1}
K^+	KCl	1.0-8.0 mmol L^{-1}	8 mmol L^{-1}
Ca^{2+}	$CaCl_2$	1.2 ± 0.0125 mmol L^{-1}	1.2 mmol L^{-1}
Mg^{2+}	$MgCl_2$	0.89 ± 0.11 mmol L^{-1}	0.9 mmol L^{-1}
Zn^{2+}	$ZnCl_2$	0.1 - 0.5 μmol L^{-1}	0.5 μmol L^{-1}
Cu^{2+}	$CuCl_2$	0.3125 μmol L^{-1}	0.3125 μmol L^{-1}
NO_2^-	$NaNO_2$	5.1 ± 0.8 μmol L^{-1}	0.01 mmol L^{-1}
Pyru	CH_3CH_2COONa	0.03 - 0.15 mmol L^{-1}	0.15 mmol L^{-1}
UA	Uric acid	5 - 25 μmol L^{-1}	25 μmol L^{-1}
AA	Ascorbic acid	100 - 200 μmol L^{-1}	200 μmol L^{-1}
Lac	Lactate	1.01 -2.09 mmol L^{-1}	2 mmol L^{-1}
Gluc	Glucose	2.5 - 4.4 mmol L^{-1}	4.4 mmol L^{-1}

DA	Dopamine Hydrochloride	30 pg mL^{-1}	30 pg mL^{-1}
Alb	Albumin	0 - 500 mg L^{-1}	500 mg L^{-1}
5HT	5-Hydroxyindole-3-acetic acid	2-3 μmol L^{-1}	3 mmol L^{-1}

Leaching test: In the leaching test, the sensor was immersed in PBS solution for 3 days and the intensity at 600 nm in 25 °C PBS solution was measured every 24 hours.

3.2 DO sensor:

DO sensor calibration: The DO sensor was characterized by mixing different proportions of 0% DO buffer solution (0 mgL^{-1}) and 100% saturated DO buffer solution to create solutions with varying degrees of oxygenation. In this study, a benchtop oxygen meter was used as the reference to measure DO values. The 100% DO buffer solution was prepared by continuously pumping concentrated air into pH 7.4 PBS solution until stabilization, while the 0% DO buffer solution involved pumping nitrogen gas until the DO reaches 0. A three-neck flak was used for sensor characterization, where one neck for benchtop oxygen meter probe, one neck for the proposed oxygen sensing fiber, and the third neck for altering DO concentrations using 0% or 100% DO solutions. The same optical measurement system was employed with 405 nm laser excitation and 660 nm emission intensity analysis.

During the experiment, DO buffer solution from 0 mg L^{-1} to 8 mg L^{-1} was prepared by adding 100% DO solution to 0 mg L^{-1} DO solution gradually. The spectrum under each DO level after stabilized was captured and used for sensitivity analysis.

Reversibility: Similar to the temperature sensor, the DO sensor's reversibility test involved dipping the sensing fiber between 0 and 8 mg/L DO buffer solutions, and emission intensity at 660 nm was recorded.

Photostability: Photostability test was carried out by exposure the DO fiber sensor to continuous 405 nm laser light for 3 hours. The intensity at 660 nm was measured continuously through this test.

Pressure dependence: Pressure dependence test was carried out in the same way as the pressure dependence test for the temperature sensor, using 100% DO saturated solution.

pH dependence: The same protocol for pH dependence test was conducted for the DO sensor as for the temperature sensor, with 100% DO saturated solution.

Temperature dependence: The temperature dependence was conducted by placing the sensing probe in 100% DO saturated solution with increasing temperature from 25-45 °C. The intensity of 660 nm at each temperature was recorded.

Leaching: The sensor was immersed in a PBS solution for four days, and the intensity at 660 nm was measured every 24 hours to calculate the leaching percentage.

Selectivity: PBS solutions with various interferents were used for the test, and the interferent concentrations are listed in Table S1. After dissolving the interferents into PBS buffer solutions, N_2 gas was bubbled to make 0% DO solutions. 100% DO solution was used to test the sensor for DO measurement.

3.3 pH sensor

pH sensor was characterized using the same way as for the other sensors. 405 nm laser was used for the sensor excitation and the intensity at 450 nm was analyzed.

Sensitivity: pH 6.0, 6.4, 6.8, 7.2, 7.6, 8.0 gradient buffer solutions were prepared using NaOH and HCl adjusted PBS solutions for the evaluation of the pH sensor sensitivity.

Reversibility: pH 6 and pH 8 PBS solutions were used for the analysis of the sensor's reversibility. The procedure was the same as for the other sensors.

Photostability: The sensor was exposed continuously to 405 nm laser source (4.5 mw) for 3 hours and the intensity at 450 nm was measured every 30 min.

Temperature dependence: The temperature dependence was tested using pH 6.8, pH 7.2 and pH 7.6 PBS solution. The temperature of the solution was heated to 35°C-41°C with an increment of 1 °C. The intensity at 450 nm was measured and correlated to the temperature.

Pressure dependence: The pressure dependence was performed using pH 7.4 PBS solution and measured using the same methods as for the other sensors.

Selectivity: In the selectivity test, each interferent was added to a glass of 300 mL pH 7.4 PBS solution and the solution was adjusted to pH 7.4 before testing. The pH sensor was dipped into the solutions and the intensity at 450 nm was recorded.

3.4 Sodium ion sensor

Fabrication: 1 mg CoroNa Green was dissolved in 1.7 mL DMSO to obtain 1 mmol L^{-1} stock solutions.

Probe concentration optimization: Probe concentration varying from 0.04-0.2 mmol·L^{-1} were prepared by diluting the stock solution. 20 μL of the diluted probe DMSO solutions were added to microplate wells and further mixed with 20 μL of 100 mmol·L^{-1} sodium tris buffer (pH=7.4) to yield 0.02-0.1 mmol·L^{-1} sodium probe concentration. Fluorescence spectra under

450 nm excitation were obtained to determine optimal probe concentration, which was then used for sodium sensor fabrication.

Sodium sensor characterization: The sodium sensor was characterized using 150 mmol L^{-1} pH 7.4 tris buffer. A 450 nm laser light was applied to the sensing film, and emission intensity at 540 nm was used for characterization.

Sensitivity: Tris buffer solutions with sodium concentration ranging from 0-200 mmol L^{-1} at 40 mmol L^{-1} increments were prepared and used. The solution was continuously stirred during measurement. Intensity at 540 nm was measured when the sensor was in each buffer and the correlation between intensity and sodium concentration was calculated.

Reversibility: The reversibility test for the sodium sensor was performed using 40 mmol L^{-1} and 140 mmol L^{-1} sodium tris buffer. The procedure was the same as for the other sensors.

Photostability: The sodium sensor was exposed to a 450 nm laser for 3 continuous hours in 140 mmol L^{-1} [Na^+] tris buffer solution. The intensity at 540 nm was measured every 15 minutes.

Pressure dependence: The pressure dependence was performed using 140 mmol L^{-1} [Na^+] buffer solution following the method used for other sensors.

Temperature dependence: The sensor was used to measure sodium free and 100 mmol L^{-1} sodium tris buffer with temperature heated from 36 – 40 °C using a hotplate.

pH dependence: Sodium free and 100 mmol L^{-1} [Na^+] tris buffer solution with pH ranging from 6-8 were prepared for the pH dependence test. The sensor was placed in these varied sodium buffer solutions and the emission intensity at 540 nm was recorded for each buffer.

Selectivity: Interferent buffer solutions were prepared with 200 mmol L^{-1} tris buffer. To maintain the same ionic strength in tests for other ions, the same ion concentrations (200 mmol L^{-1}) were added to the tris buffer solution. For metabolites, concentrations were as in Table S1. Sensor emission intensity when measuring these buffer solutions was recorded and normalized to the blank solution (pure tris buffer).

3.5 Calcium sensor

Fabrication: 0.5 mg calcium fluorescence probe Rhod-5N was dissolved in 0.556 mL DI water to obtain 1 mmol L^{-1} stock solutions.

Probe concentration optimization: Probe concentration varying from 0.04-0.2 mmol·L^{-1} with increments of 0.02 mmol·L^{-1} were prepared by diluting the stock solution. 20 μL of the diluted probe solution were added to microplate wells and further mixed with 20 μL of 1 mmol·L^{-1} calcium tris buffer (pH=7.4) to results 0.02-0.1 mmol·L^{-1} calcium probe

concentration. The fluorescence intensity at 580 nm excited with 520 nm light were obtained to find the optimal probe concentration.

PolyAM hydrogel optimization: Due to the calcium sensor is water soluble, the formulation of the polyacrylamide film was optimized to reduce the leaching of the calcium probe. PAM films with different monomer concentration (w/v%: 25-40%) and crosslinker concentration (w/v% 0-10%) were fabricated and the leaching of the film after 3 days was used to tailor the monomer and crosslinker concentrations.

Once the probe fabrication method was optimized, calcium sensing film fabrication and characterization studies were conducted. Same as sodium sensor, calcium sensor was also characterized using 150 mmol L^{-1} tris buffer. A 520 nm laser was used for the excitation of the sensing film.

Sensitivity: Calcium tris buffer solutions were prepared by dissolving $CaCl_2$ into 150 mmol L^{-1} tris buffer solution. 0, 0.25, 0.5, 1.0, 1.5, and 2.0 mmol L^{-1} Ca^{2+} gradient buffer solutions were used for the sensitivity measurement. The sensor was submerged into the solutions for the measurement and the emission spectrum of each solution was recorded. The emission intensity with the corresponding calcium ion concentration was analyzed.

Reversibility: The reversibility was evaluated using 0.25 mmol L^{-1} and 1 mmol L^{-1} calcium tris buffer solutions. The same procedure was followed as for the other sensors.

Photostability: The sensor was excited under the exposure of 520 nm laser light for a continuous of 180 min. The 540 nm emission intensity was recorded every 15 min.

Pressure, pH, and temperature dependence: The pressure, pH and temperature dependence tests were measured using the same way as the sodium sensor. Instead, here calcium free and 1 mmol L^{-1} calcium buffer solution was used.

Selectivity: The same interferent solution as in the sodium sensor selectivity test was used here. However, for calcium sensor, 1 mmol L^{-1} concentration was used for the ion selectivity test. The procedures were the same as the other sensors.

3.6 Glucose Sensor

Fabrication: The same optical setup was used for glucose sensor characterization. A 520 nm laser (4.5 mW) was used for sensor excitation and emission intensity at 640 nm was recorded for following analysis. The buffer solutions were prepared using pH 7.4 PBS solution and the experiments were conducted under room temperature.

Sensitivity: The glucose sensor's sensitivity was characterized using 0-6 mmol L^{-1} glucose PBS solution (pH = 7.4) with 1 mmol L^{-1} of increment. The sensor's spectrum of each buffer

solution was recorded and the emission intensity at 640 nm was correlated to the glucose concentration to obtain the calibration curve of the sensor.

Reversibility: The reversibility test was performed using 1 mmol L^{-1} and 5 mmol L^{-1} glucose buffer solutions. The sensor was dipped reversely in between the two buffer solutions and the intensity at 640 nm was used to indicate sensor reversibility.

Pressure dependence: The pressure dependence was the same as for the other sensors but instead 4 mmol L^{-1} glucose pH=7.4 PBS solution was used here.

Photostability: The glucose sensor was continuously excited using 520 nm laser for 3 hours with the sensor immerged in 4 mmol L^{-1} glucose PBS solution. The intensity at 640 nm was recorded every 30 min.

pH dependence: The enzyme activity is pH dependent. A lower activity may lead to a low emission intensity due to decreased ratio of oxygen consumption speed to oxygen supply speed. In the pH dependence test, 4 mmol L^{-1} glucose PBS solution was adjusted to a series of pH 6-8 4 mmol L^{-1} glucose buffer. The emission intensity of each solution was measured for analysis of pH dependence.

Temperature dependence: Glucose-free and 1 mmol L^{-1} glucose PBS solution was used for temperature dependence test. The temperature of the solution was controlled to 34-41 ℃ using a hotplate.

Selectivity: Major interferents were dissolved in PBS solutions separately at specific concentration (**Table S1**). The pH of the interferent solutions was adjusted to 7.4 before testing. During the test, the glucose sensor was dipped into the interferent solutions sequentially and the emission intensity at 640 nm was recorded and normalized to blank solution.

4. Multiplexed sensor characterization

After the 6 sensors were characterized individually, the optical fiber sensors were bundled together and characterized for multiplexed sensing using prepared aCSF buffer solutions. aCSF was prepared by dissolving KCl, KH_2PO_4, $NaHCO_3$, and $MgCl_2$, in DI water and the concentration of them were 1.0 mmol L^{-1}, 1.2 mmol L^{-1}, and 26 mmol L^{-1} respectively. The temperature, DO, pH, Na^+, Ca^{2+}, and glucose concentrations of the aCSF solutions were prepared as described in Table S2. The temperature of the solution was controlled using a hot plate. The DO was controlled by pumping nitrogen into the solution. The pH was altered by dipping HCl or NaOH to the solution. The [Na^+], [Ca^{2+}], and glucose level were controlled by dissolving different amount of NaCl, $CaCl_2$, and Glucose. A series of readings of the sensors' intensities under gradient biomarker concentrations (**Table S2**) were recorded and the

correlations between the spectrum and the biomarker concentration would be used to validate the sensor's performance. The spectra after the signal stabilized were recorded and used for calibration. The experiment was repeated three times.

Table S2. Buffer solution preparation for multiplexed sensor characterization

	T (°C)	DO (mg L^{-1})	pH	Na^+(mmol L^{-1})	Ca^{2+}(mmol L^{-1})	Gluc(mmol L^{-1})
T buffer	36-40	8	7.4	140	0.5	2
DO buffer	37	0-8	7.4	140	0.5	2
pH buffer	37	8	6-8	140	0.5	2
Na^+ buffer	37	8	7.4	100-180	0.5	2
Ca^{2+} buffer	37	8	7.4	140	0, 0.25, 0.5, 1, 2	2
Glu buffer	37	8	7.4	140	0.5	0, 1, 2, 4, 6

During the test, the designed fiber bundle was dipped into the solution and excited with corresponding laser light. The spectra after the signal stabilized were recorded and used for calibration. The experiment was repeated three times.

5. Signal post processing and machine learning modeling of the obtained spectra

To realize online continuous readout of these 6 brain biomarkers simultaneously, machine learning models were trained and built to realize accurate readout based on the obtained spectra. In this study, three spectra are obtained in each scan, they are fluorescent spectrum including the information of pH, glucose, and DO under the excitation of 405 nm laser light, fluorescent spectrum for the sodium ion and temperature sensors under the excitation of 488 nm laser light, and the fluorescent spectrum for the readout of calcium ion under the excitation of 520 nm laser light.

5.1 Dataset:

The dataset was obtained by placing the multiplexed fiber bundle in standard PBS buffer solutions with varied biomarker concentrations. Each obtained spectrum was labelled as the biomarker concentrations of the standard buffer solution. A total dataset of 18875 spectra, containing different combination of the 6 fluorescence sensors under various concentrations were obtained for model training and validation. The total exposure time until the capturing of each spectrum was also recorded for photobleaching correction.

5.2 Data preprocessing

Before feeding into the machine models for model training, the spectra were first pre-processed to remove the irrelevant information and background noise. The baselines of the spectra were also corrected to eliminate the background effects. Then the processed spectra were merged into one spectrum for the model input.

5.3 Data smoothing

After obtaining the spectrum, Savitzky-Golay filter was used to smooth the spectrum, which has been frequently employed for signal processing. The filter is capable of both removing high-frequency noises and attenuating low-frequency components of a signal. The algorithm is based on the polynomial fitting of the spectrum and here a polynomial order of 3 was used to correctly smooth the signal while avoiding peak shifts.

5.4 Baseline correction algorithms

Define the measured spectrum vector as $\mathbf{X}=[\boldsymbol{x_1}, \boldsymbol{x_2}, \dots, \boldsymbol{x_m}]^{\mathbf{T}}$, and the corrected spectrum vector as $\mathbf{Z}=[\boldsymbol{z_1}, \boldsymbol{z_2}, \dots, \boldsymbol{z_m}]^{\mathbf{T}}$, where m is the spectrum length.

Fidelity of **Z** to **X** is defined as:

$$F = \sum_{i=1}^{m} \boldsymbol{w_i}(\boldsymbol{x_i} - \boldsymbol{z_i})^{\mathbf{2}} \quad \text{(Eq. S2)}$$

, where w_i is the weight of fidelity to each spectrum value at different wavelengths.

The roughness of Z is defined as:

$$R = \sum_{i=1}^{m-1}(\boldsymbol{z_{i+1}} - \boldsymbol{z_i})^{\mathbf{2}} = \sum_{i=1}^{m-1}(\boldsymbol{\Delta z_i})^{\mathbf{2}} \quad \text{(Eq. S3)}$$

To balance the fidelity and roughness of the fitted spectrum, we defined a combined equation as Q:

$$Q = F + \lambda R = \boldsymbol{W}||\boldsymbol{X} - \boldsymbol{Z}||^2 + \lambda||\boldsymbol{\Delta Z}||^2 \quad \text{(Eq. S4)}$$

, where **DZ**= $\boldsymbol{\Delta}$**Z**, and λ can be adjusted manually. The larger the λ, the smoother the corrected spectrum.

Therefore, the aim of the baseline correction is to minimize Q, which can be achieved by calculating the partial derivatives of Q and make it equal to 0 ($\frac{\partial Q}{\partial z} = 0$).

Therefore,

$$\boldsymbol{Z} = (\boldsymbol{W} + \lambda \boldsymbol{D'D})^{-1}\boldsymbol{WX} \quad \text{(Eq. S5)}$$

The weights were iteratively adjusted based on the location of each data at the spectrum. For example, at the peak of the data, the weight should be set as zero so that it wouldn't be smoothed by the algorithm. Therefore, in the iteration, the weights were updated as following:

$$w_i^t = \begin{cases} 0, \ if\ \boldsymbol{x_i} \geq z_i^{t-1}\ (peak) \\ e^{\frac{t(x_i - z_i^{t-1})}{|d^t|}}, if\ \boldsymbol{x_i} < z_i^{t-1}\ (non-peak) \end{cases} \quad \text{(Eq. S6)}$$

, where t is the number of iterations, and d^t denotes the differences between **X** and $\mathbf{Z}^{t-1}$ in the current (t) iteration. The iteration stops when the iteration number is reached or when $|\mathbf{d}^t| < 0.001\times|\mathbf{X}|$.

A Python program is implemented to iteratively adapt the fidelity rate and calculate the corrected spectrum.

5.5 Data merging

Each spectrum obtained contains 741 intensity values, and the independently obtained three spectra of three laser lights are merged into 1 dataset by simply stacking them along the axis to obtain 2,223 intensity values in each set. It is noticed that 842 intensity values are irrelevant to the biomarker concentrations as these values stay the same in every dependent variable, which were then removed from the dataset. Therefore, a total of 1,381 values were utilized in each scan of the 6 sensors.

5.6 Machine learning modeling

Linear Regression: Linear regression models the relationship between a dependent variable and one or more independent variables by fitting a linear equation to the data. Simple linear regression has one independent variable, while multiple linear regression handles multiple predictors.

Lasso Regression (L1 Regularization): Lasso (Least Absolute Shrinkage and Selection Operator) is a linear regression technique that adds a penalty term based on the absolute values of the coefficients. Lasso can be used for feature selection and helps prevent overfitting by encouraging some coefficients to be exactly zero, effectively eliminating irrelevant features.

Ridge Regression (L2 Regularization): Ridge regression is another form of linear regression with a penalty term, but this time based on the squared values of the coefficients. It's used to mitigate multicollinearity (correlations between independent variables) and reduce the impact of large coefficient values. Ridge doesn't force coefficients to zero, but it shrinks them.

Bayesian Regression: Bayesian regression is a framework that applies Bayesian statistics to linear regression. It estimates the posterior distribution of model parameters, allowing for uncertainty quantification. Bayesian regression is useful when you want to incorporate prior knowledge or have a better understanding of parameter uncertainty. It provides a distribution of possible parameter values instead of a single point estimate.

Hyperparameter fine tuning:

Grid search was applied to find the best parameters, which searches for the best hyperparameters comprehensively by analysing all possible combinations. The hyperparameters that needed to be tuned are displayed in **Table S3**.

Table S3. Tuned hyperparameter list for regression models

Model	Hyperparameter Lists
Linear	n_jobs
Ridge	alpha
Lasso	alpha
Bayesian	alpha_1, alpha_2, lambda_1, lambda_2

6. System validation using ex vivo brain model

The proposed system's capability to identify secondary brain injuries in TBI patients was tested using lamb brain models to simulate three TBI complications: hypoxia, hypermetabolism, and excitotoxicity. Hypoxia involves high temperature and oxygen deficiency, hypermetabolism includes low glucose levels along with hypoxia symptoms, and excitotoxicity leads to increased acidity, sodium, and calcium ion levels in the brain.

To mimic these scenarios, corresponding buffer solutions were prepared and introduced into clean lamb brains. The developed fiber bundle sensors, integrated with a medical-grade catheter, were inserted into the brain tissues during the process. Transitions between scenarios were achieved by changing buffer solutions, and spectra were captured every 3 seconds using a python program. Lasers were automatically switched between 405 nm, 488 nm, and 520 nm for different sensor excitations and biomarker measurements. Simulated transitions from complications to a healthy state were also conducted to replicate disease recovery situations. Signal processing employed the proposed algorithms for peak separation, environmental compensation, and biomarker concentration calculation.

Table S4. aCSF buffer solution preparation for different brain scenarios

	T (°C)	DO (mg L^{-1})	pH	Na^+(mmol L^{-1})	Ca^{2+}(mmol L^{-1})	Gluc(mmol L^{-1})
Hypoxia	38	3	7.5	140	1.5	2.5
Hypermetabolism	39	3	7.2	130	1.2	1
Excitotoxicity	37	7	6.4	180	2	3
Normal1	36	7	7.5	140	1.5	2.5

Normal2	37.5	7	7.2	140	1.5	3

7. Human CSF measurements

7.1 Patient recruitment:

Patients with a fractured neck of femur emergent admitted to St Mary's Hospital, London, UK, were considered for recruitment over an 18-month period.

7.2 Exclusion criteria:

Recent use of antiplatelet or anticoagulant medication; Previous lumbar spine surgery; Previous history of post-dural puncture headache; Body mass index > 30 kg/m^2; History of alcohol or drug abuse; Inability to consent to surgery; Pre-existing cognitive impairment or Parkinson's Disease; Psychotropic or corticosteroid medication use.

7.3 Experimental protocol:

The developed multiplexed optical bundle sensors were further validated using clinical human CSF samples to examine its ability in reflecting biomarker concentration variations in real CSF samples. Before the spiking test, the sensing system was first calibrated using two standard buffer solutions. The sensors' spectra of the standard buffer solutions were saved for sensing readout correction.

Table S5. Calibration buffer solutions

	T (°C)	DO (mg L^{-1})	pH	Na^+(mmol L^{-1})	Ca^{2+}(mmol L^{-1})	Gluc(mmol L^{-1})
Buffer solution 1	25	8	8	140	0	0
Buffer solution 2	40	0	6	200	2	6

7.3.1 DO testing:

10 mL human CSF sample was used for the DO sensor validation. A three-neck flask was used. The DO was adjusted to 1,3,6,8 mg/L according to the reading of the DO meter. The DO sensor was excited using 405 nm laser light continuously throughout the experiment and the spectrum was obtained every 3 second and labelled according to the DO meter reading.

7.3.2 Temperature testing:

The same 20 mL CSF sample from DO test was used for the temperature sensor validation. The temperature of the sample was adjusted to 34, 36, 38, 40 °C using a hotplate and monitored with a thermometer. The temperature sensor was excited using a 450 nm laser light

continuously throughout the experiment and the spectrum was obtained every 3 second and labelled according to the thermometer reading.

7.3.3 pH testing:

After temperature sensor testing, the sample was used for the pH sensor validation. During the experiment, both the designed multiplexed bundle sensor and pH meter (reference) were inserted into the sample. For the pH measurement, 405 nm laser light was used. The light and spectrometer worked in continuous mode and the spectrum of the sensors was taken every 3 seconds. HCl and NaOH were used to adjust the pH of the sample gradually to 6.5, 7.0, 7.5, and 8.0 in consecutive, and once the pH meter reading stabilized the pH probe was inserted for the measurement. Then the buffer was adjusted to the next level based on the reading of the pH meter.

7.3.4 Sodium ion testing:

10 mL of the diluted human CSF sample was used for the sodium sensor validation. Due to the fact that the existing sodium ion concentration was unknown in the sample, we measured the sensor's performance using NaCl spiked sample. The sodium sensor was excited using 450 nm laser light continuously throughout the experiment and the spectrum was obtained every 3 seconds. When the signal stabilized, an additional 11.7 mg NaCl was added to the sample with constant stirring to obtain 20 mmol L^{-1} NaCl spike. In total 60 mmol L^{-1} of NaCl spike was added to the sample in three additions.

7.3.5 Calcium ion testing:

30 mL of the diluted human CSF sample was used for the calcium sensor validation. The sensor's performance was measured using $CaCl_2$-spiked sample. The calcium sensor was excited using a 520 nm laser light continuously throughout the experiment and the spectrum was obtained every 3 seconds. When the signal stabilized, an additional 1.665 mg $CaCl_2$ was added to the sample with constant stirring to obtain 0.5 mmol L^{-1} of $CaCl_2$ spike. In total 1.5 mmol L^{-1} of $CaCl_2$ spike was added in three additions.

7.3.6 Glucose testing:

10 mL of the diluted human CSF sample was used for the glucose sensor validation. The sensor's performance was evaluated using D-(+)-glucose-spiked sample. The glucose sensor was excited using a 405 nm laser light continuously throughout the experiment and the spectrum was obtained every 3 seconds. When the signal stabilized, an additional 2 mmol L^{-1} D-(+)-glucose (4.8 mg) was added to the sample with constant stirring. In total 6 mmol L^{-1} of D-(+)-glucose was added to the sample in three additions.

Each test was repeated three times and the calculated concentrations of each biomarker after environmental compensation were recorded in continuous mode and saved for system evaluation. For pH, DO, and temperature measurement, MSE and MAE were used to evaluate the system's performance. For Na^{+}, Ca^{2+}, and glucose, recovery rate was used for the evaluation.

Results

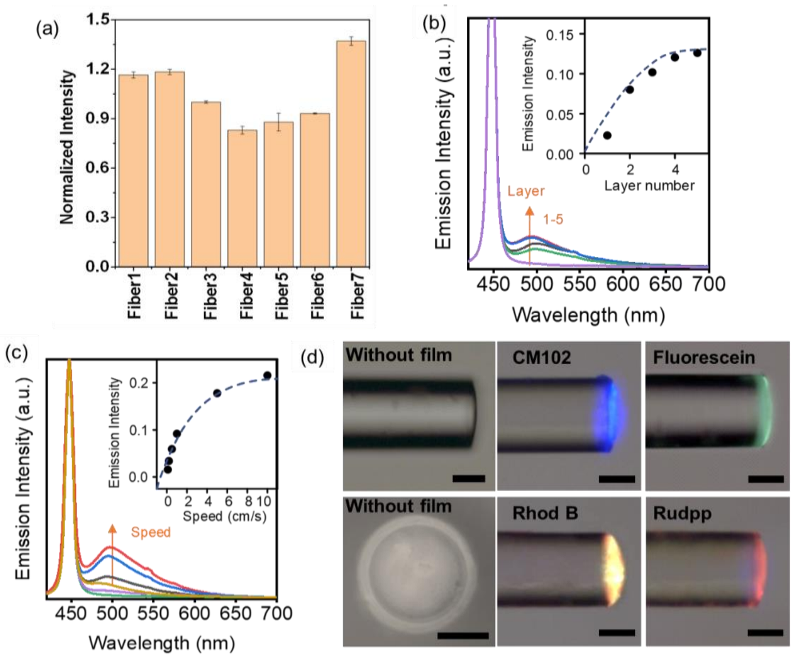

Figure S2. Optimization of bundle fabrication and dip coating speed and layers. (a) Light transmission efficiency comparison of the 7 fibers of the bundle. (b) Fluorescent emission spectra with different coating layers. Inset: Correlation between dip coating layer numbers and film intensity. (c) Fluorescent emission spectra under different coating speed. Inset: Correlation between dip coating speed and film intensity. (d) Fluorescent film coatings on fiber tips. Scale bar= 50 µm. n=3.

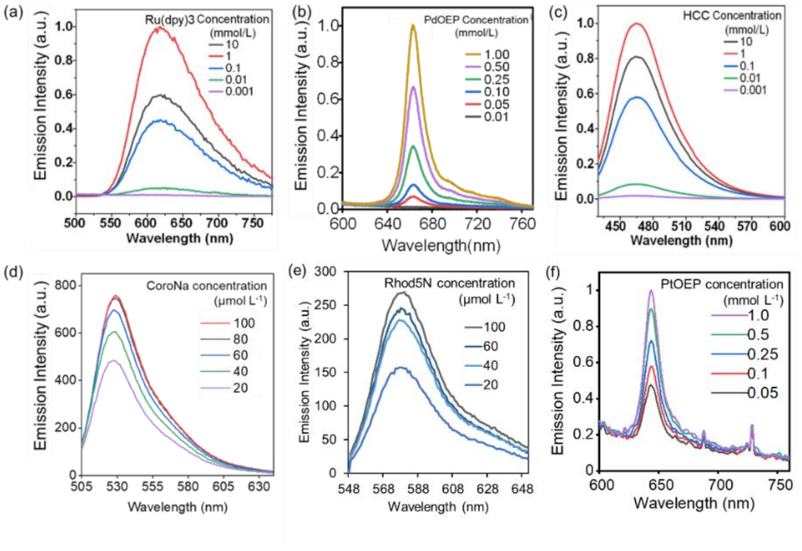

Figure S3. Probe concentration optimization for (a) Ru(dpy)$_3$, (b) PdOEP, (c) HCC, (d) CoroNa, (e) Rhod5n, (f) PtOEP.

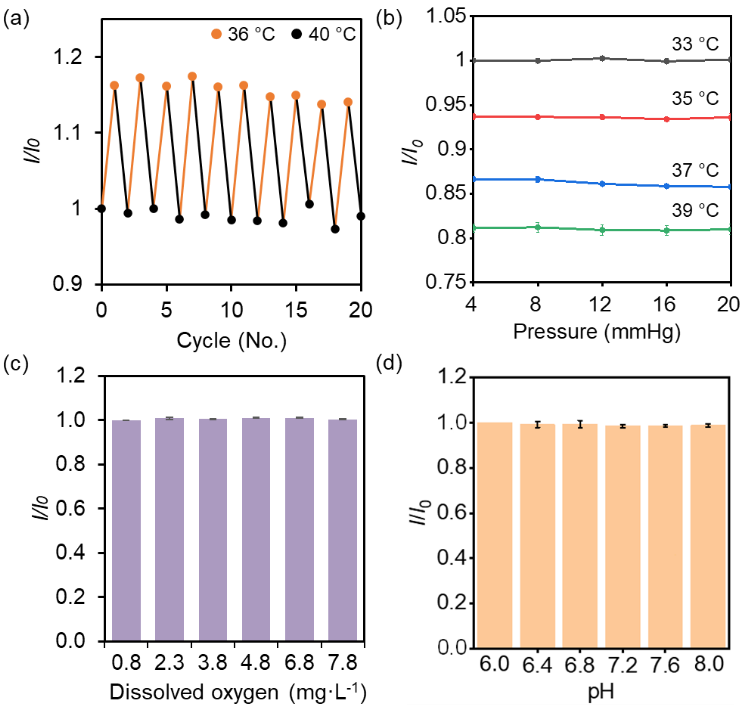

Figure S4. Characterization of temperature sensor. (a) Reversibility of the sensor for repeated measurements in PBS solutions at 36 and 40°C. (b) Pressure dependence of the sensor in 37°C PBS with 4-20 mmHg pressure. (v) Oxygen dependence test of the temperature sensor in 37°C PBS solution with varying DO (0-6 mg L^{-1}) values. (d) pH dependence test of the sensor in 37°C PBS solution with 6.0-8.0 pH levels. n=3.

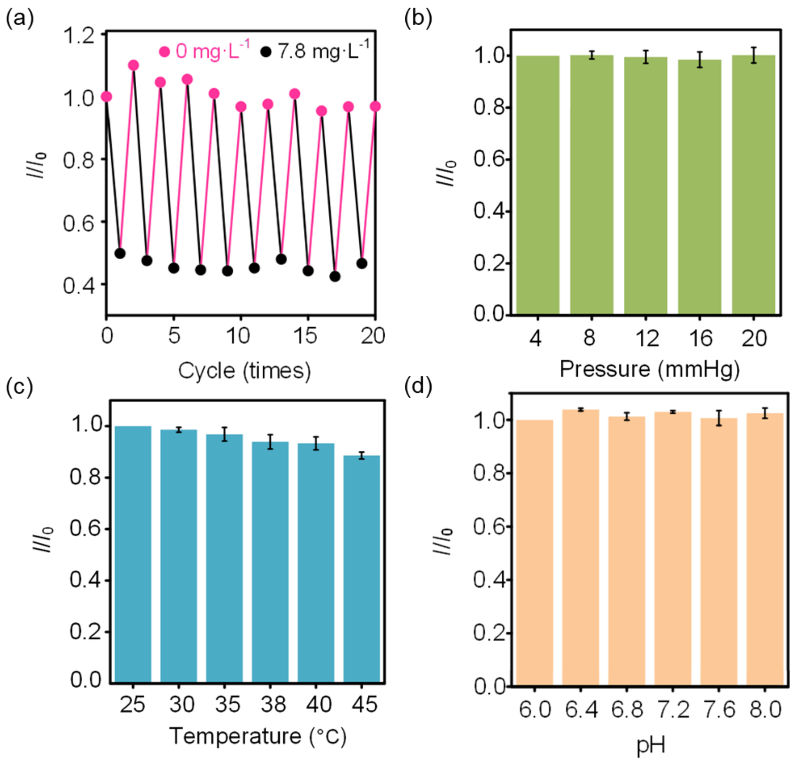

Figure S5. Characterization of the DO sensor. (a) Reversibility test of the DO sensor in the repeated measurement of 0 and 7.8 mg L-1 DO PBS solution. (b) Pressure dependence of the DO sensor in 7.8 mg L^{-1} DO PBS solution with pressure from 4-20 mmHg. (c) Temperature dependence test of the sensor using 25-45 °C 7.8 mg L^{-1} DO PBS solution. (d) pH dependence tests of the DO sensor in 7.8 mg L^{-1} DO PBS solution with pH from 6.0-8.0. n=3.

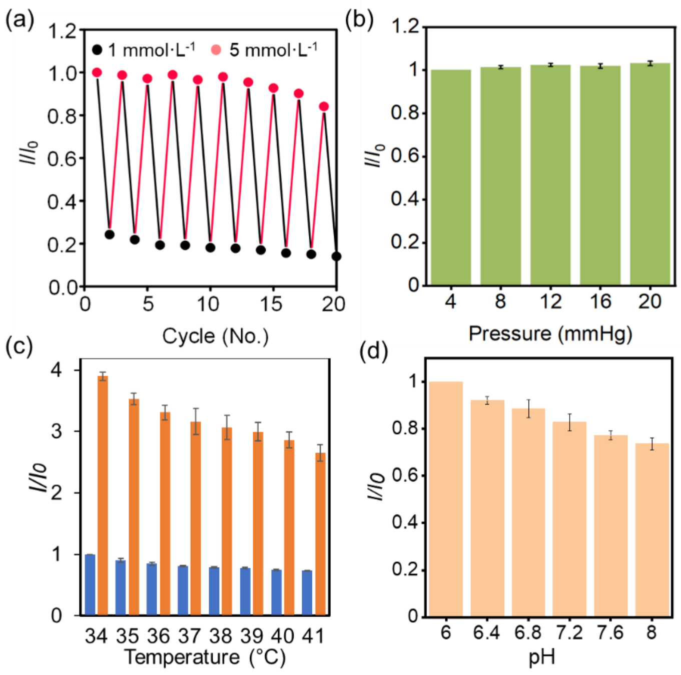

Figure S6. Characterization of the Glucose sensor. (a) Reversibility test of the glucose sensor in repeated measurement of 1 and 5 mmol L^{-1} glucose PBS solution. (b) Pressure dependence test using 2 mmol L^{-1} glucose solution with pressure increasing from 4 to 20 mmHg. (c) Temperature dependence test using 0 and 4 mmol L^{-1} glucose PBS solution with temperature increasing from 34 to 41°C. (d) pH dependence test using 2 mmol L^{-1} glucose solution with pH from 6 to 8. n=3.

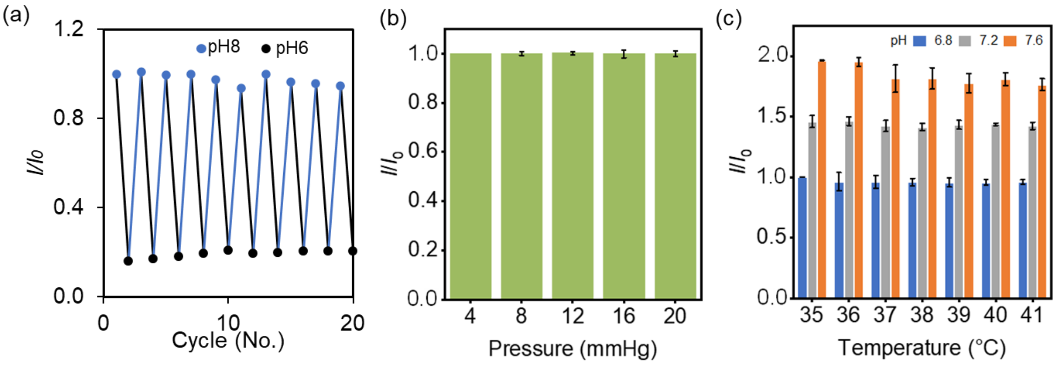

Figure S7. Characterization of the pH sensor. (a) Reversibility test of the sensor in sensing pH 6.0 and 8.0 PBS solutions. (b) Pressure dependence test using pH 7.4 PBS solution with pressure increasing from 4 to 20 mmHg. (c) A temperature dependence test using pH 7.4 PBS solution with temperature increasing from 35°C to 41°C. n=3.

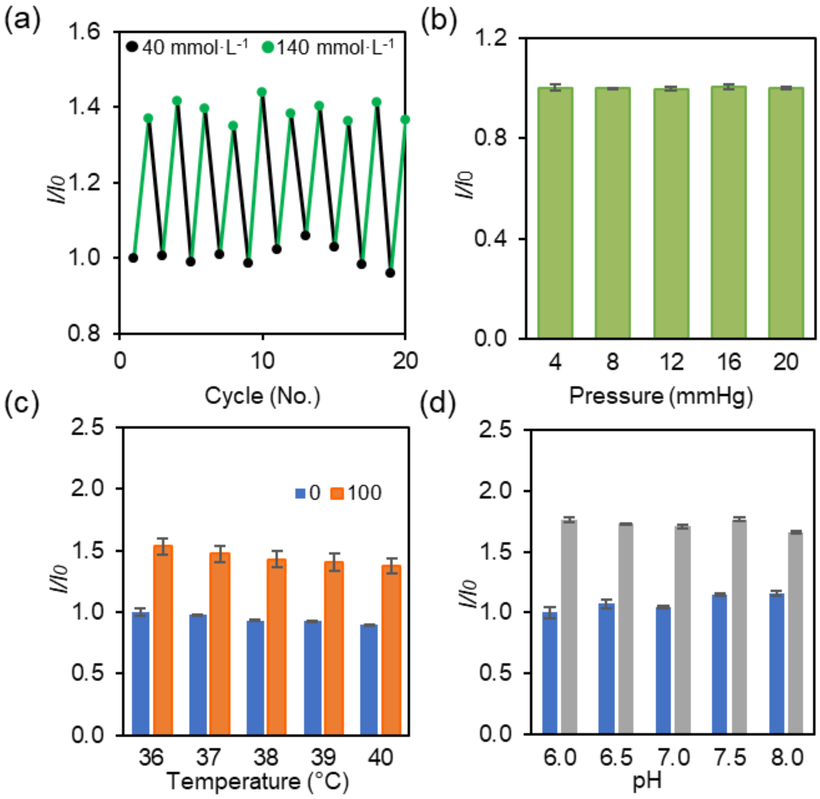

Figure S8. Characterization of the Na^+ sensor. (a) Reversibility of the Na^+ sensor for repeated measurement of 40 and 140 mmol L^{-1} [Na^+] Tris buffer solution. (b) Pressure dependence test

using 140 mmol L^{-1} [Na^+] Tris buffer solution with pressure from 4-20 mmHg. (c) Temperature dependence test using Na^+-free and 100 mmol L^{-1} [Na^+] Tris buffer solution with temperature from 36 to 40 °C. (d) pH dependence tests using Na^+-free and 100 mmol L^{-1} [Na^+] Tris buffer solution with pH from 6.0 to 8.0. n=3.

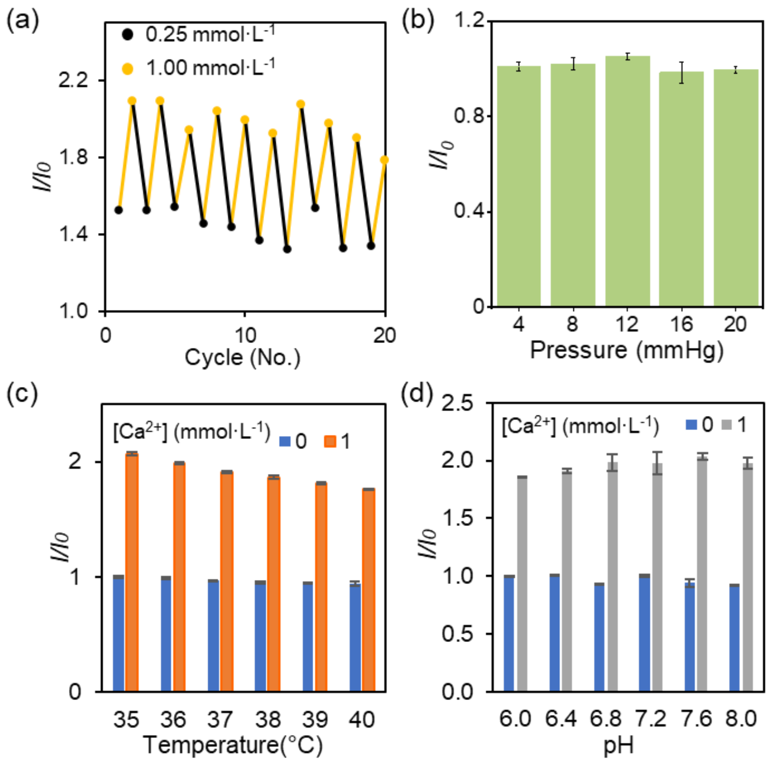

Figure S9. Characterization of the Ca^{2+} sensor. (a) Reversibility of the Ca^{2+} sensor for repeated measurement of 0.25 and 1 mmol L^{-1} [Ca^{2+}] Tris buffer solution. (e) Photostability of the Ca^{2+} ion sensor under continuous exposure to 520 nm laser light (4.5 mW) for 2 hours. (b) Pressure dependence test using 1.0 mmol L^{-1} [Ca^{2+}] Tris buffer solution with pressure from 4-20 mmHg. (c) Temperature dependence test using Ca^{2+} free and 1 mmol L^{-1} [Ca^{2+}] Tris buffer solution with temperature from 36 to 40 °C. (d) pH dependence test using Ca^{2+}-free and 1 mmol L^{-1} [Ca^{2+}] Tris buffer solution with pH from 6.0 to 8.0. n=3.

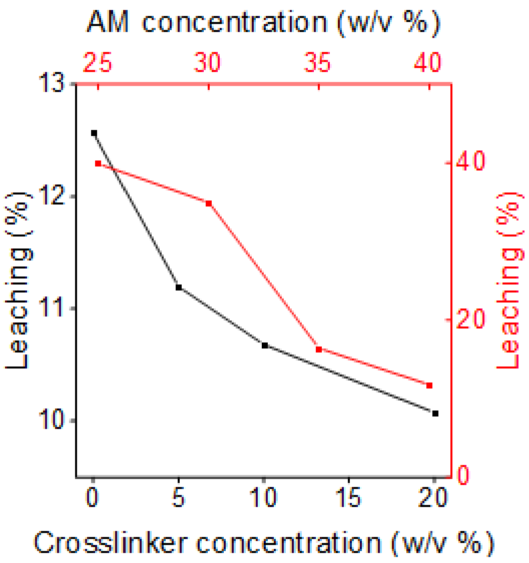

Figure S10. Correlation between crosslinker, AM, and leaching ratios for calcium ion sensing film fabrication.

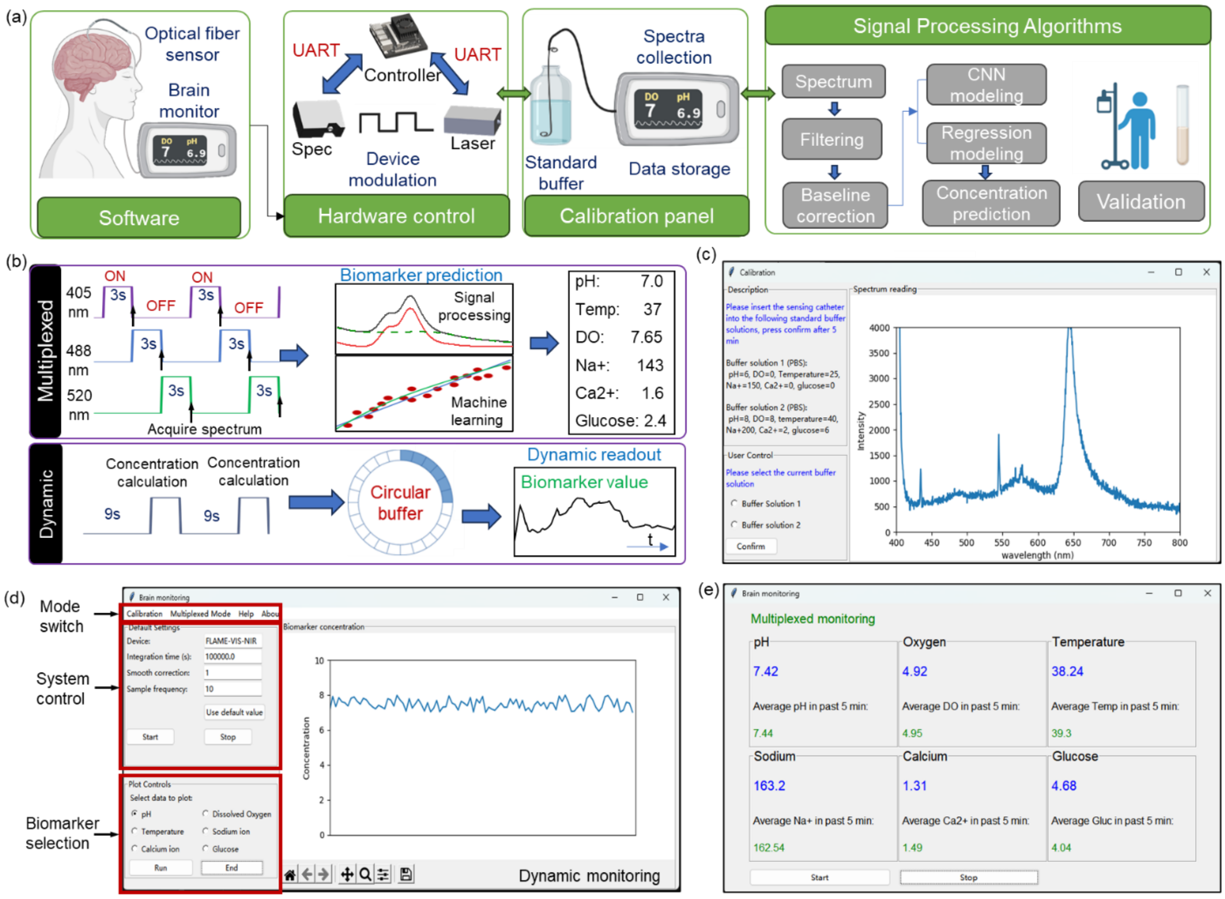

Figure S11. Software design for continuous brain monitoring. (a) The structure and realization of the software. (b) Multiplexed and dynamic modes design and methods. (c) Screenshot of the calibration panel of the software. (d) Screenshot of the initiation frame of

the software which is also used for device control and biomarker dynamic monitoring. (e) Screenshot of the multiplexed monitoring of 6 brain biomarkers simultaneously.

Table S6. Biomarker concentration calculation comparison between various machine learning regression models.

		Training			Validation			Testing		
Models	Biomarker	R^2	MSE	MAE	R^2	MSE	MAE	R^2	MSE	MAE
Bayesian	pH	0.98	0.1	0.03	0.99	0.07	0.05	0.99	0.05	0.04
	DO	0.91	0.82	0.59	0.92	0.77	0.59	0.94	0.6	0.47
	Temperature	0.97	0.27	0.12	0.98	0.2	0.12	0.99	0.12	0.1
	Sodium	1	1.85	1.31	1	1.87	1.32	1	2.05	1.5
	Calcium	0.95	0.16	0.09	0.95	0.17	0.1	0.95	0.18	0.11
	Glucose	0.93	0.42	0.29	0.94	0.38	0.29	0.96	0.35	0.27
Lasso	pH	-0.02	0.76	0.67	1	0.04	0.03	1	0.03	0.02
	DO	0.74	1.41	1.24	0.94	0.68	0.53	0.95	0.54	0.42
	Temperature	0.25	1.25	1.1	0.99	0.15	0.12	0.99	0.13	0.12
	Sodium	0.99	4.19	3.15	1	1.83	1.34	1	2.04	1.52
	Calcium	-0.01	0.74	0.66	0.95	0.17	0.1	0.95	0.18	0.11
	Glucose	0.52	1.1	0.9	0.96	0.33	0.27	0.96	0.35	0.29
Linear	pH	0.98	0.1	0.03	0.98	0.1	0.03	1	0.02	0.01
	DO	0.67	1.59	0.52	0.67	1.59	0.52	0.96	0.51	0.42
	Temperature	0.97	0.25	0.12	0.97	0.25	0.12	0.99	0.12	0.09
	Sodium	0.99	2.69	1.51	0.99	2.69	1.51	1	2.07	1.46
	Calcium	0.93	0.2	0.1	0.93	0.2	0.1	0.94	0.19	0.11
	Glucose	0.88	0.56	0.29	0.88	0.56	0.29	0.95	0.39	0.31
Ridge	pH	0.99	0.08	0.04	0.99	0.07	0.05	0.99	0.06	0.04
	DO	0.91	0.82	0.58	0.92	0.76	0.6	0.93	0.64	0.5
	Temperature	0.99	0.17	0.13	0.98	0.19	0.15	0.99	0.16	0.13
	Sodium	0.99	3.78	2.65	0.98	4.38	3.22	0.99	3.3	2.3
	Calcium	0.95	0.16	0.08	0.95	0.17	0.1	0.94	0.18	0.12
	Glucose	0.93	0.42	0.29	0.95	0.34	0.28	0.96	0.37	0.29

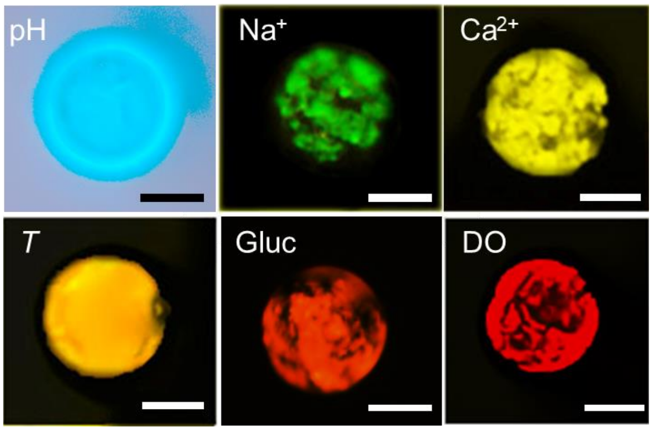

Figure S12. Fluorescent photographs of the 6 sensing films on the tips of the fiber bundle.

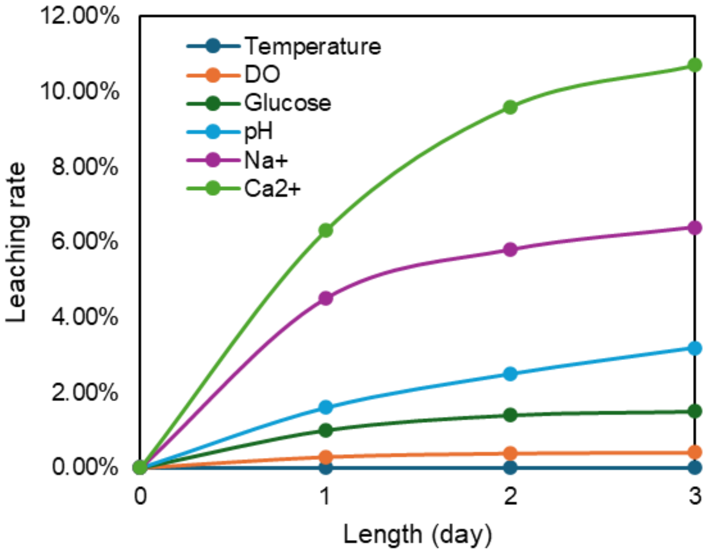

Figure S13. Leaching rates of the six sensors after 3 days of immersion in PBS solutions.
